# Engaging Children and Young People in Digital Mental Health Interventions: Systematic Review of Modes of Delivery, Facilitators, and Barriers

**DOI:** 10.2196/16317

**Published:** 2020-06-23

**Authors:** Shaun Liverpool, Catarina Pinheiro Mota, Célia M D Sales, Anja Čuš, Sara Carletto, Camellia Hancheva, Sónia Sousa, Sonia Conejo Cerón, Patricia Moreno-Peral, Giada Pietrabissa, Bettina Moltrecht, Randi Ulberg, Nuno Ferreira, Julian Edbrooke-Childs

**Affiliations:** 1 Evidence-Based Practice Unit University College London and Anna Freud National Centre for Children and Families London United Kingdom; 2 Center for Psychology University of Porto Porto Portugal; 3 University of Trás-os-Montes and Alto Douro Porto Portugal; 4 Faculty of Psychology and Education Sciences University of Porto Porto Portugal; 5 Department of Child and Adolescent Psychiatry Medical University of Vienna Vienna Austria; 6 Department of Clinical and Biological Sciences University of Turin Turin Italy; 7 Faculty of Philosophy, General, Experimental, Developmental, and Health Psychology Sofia University Sofia Bulgaria; 8 School of Digital Technologies Tallinn University Tallinn Estonia; 9 Biomedical Research Institute of Malaga Málaga Spain; 10 Department of Psychology Catholic University of Milan Milan Italy; 11 Psychology Research Laboratory IRCCS Istituto Auxologico Italiano Milan Italy; 12 Division of Mental Health and Addiction University of Oslo Oslo Norway; 13 University of Nicosia Nicosia Cyprus

**Keywords:** mHealth, eHealth, technology, smartphone, children, adolescent mental health, mobile phone

## Abstract

**Background:**

There is a high prevalence of children and young people (CYP) experiencing mental health (MH) problems. Owing to accessibility, affordability, and scalability, an increasing number of digital health interventions (DHIs) have been developed and incorporated into MH treatment. Studies have shown the potential of DHIs to improve MH outcomes. However, the modes of delivery used to engage CYP in digital MH interventions may differ, with implications for the extent to which findings pertain to the level of engagement with the DHI. Knowledge of the various modalities could aid in the development of interventions that are acceptable and feasible.

**Objective:**

This review aimed to (1) identify modes of delivery used in CYP digital MH interventions, (2) explore influencing factors to usage and implementation, and (3) investigate ways in which the interventions have been evaluated and whether CYP engage in DHIs.

**Methods:**

A literature search was performed in the Cochrane Library, Excerpta Medica dataBASE (EMBASE), Medical Literature Analysis and Retrieval System Online (MEDLINE), and PsycINFO databases using 3 key concepts “child and adolescent mental health,” “digital intervention,” and “engagement.” Preferred Reporting Items for Systematic Reviews and Meta-Analyses guidelines were followed using rigorous inclusion criteria and screening by at least two reviewers. The selected articles were assessed for quality using the mixed methods appraisal tool, and data were extracted to address the review aims. Data aggregation and synthesis were conducted and presented as descriptive numerical summaries and a narrative synthesis, respectively.

**Results:**

This study identified 6 modes of delivery from 83 articles and 71 interventions for engaging CYP: (1) websites, (2) games and computer-assisted programs, (3) apps, (4) robots and digital devices, (5) virtual reality, and (6) mobile text messaging. Overall, 2 themes emerged highlighting intervention-specific and person-specific barriers and facilitators to CYP’s engagement. These themes encompassed factors such as suitability, usability, and acceptability of the DHIs and motivation, capability, and opportunity for the CYP using DHIs. The literature highlighted that CYP prefer DHIs with features such as videos, limited text, ability to personalize, ability to connect with others, and options to receive text message reminders. The findings of this review suggest a high average retention rate of 79% in studies involving various DHIs.

**Conclusions:**

The development of DHIs is increasing and may be of interest to CYP, particularly in the area of MH treatment. With continuous technological advancements, it is important to know which modalities may increase engagement and help CYP who are facing MH problems. This review identified the existing modalities and highlighted the influencing factors from the perspective of CYP. This knowledge provides information that can be used to design and evaluate new interventions and offers important theoretical insights into how and why CYP engage in DHIs.

## Introduction

### Prevalence of Mental Health Problems in Children and Young People

Mental health (MH) problems in childhood and adolescence are of great importance because of their prevalence, early onset, and impact on different areas of the child’s life [1]. The number of children and young people (CYP) who experience MH problems ranges from 10% to 20% worldwide [2]. An international study conducted in 27 countries estimated the worldwide pooled prevalence of MH problems to be 13.4% among CYP [3]. Specifically, anxiety and disruptive behavior disorders seem to be the most frequent presentations (Table 1) [3]. Estimates further suggest that approximately 1 in every 3 adolescents will meet the criteria for anxiety and depressive disorders [2], while 1 in 4 young people aged 16 to 24 years has experienced at least one MH problem in the past year [3].

**Table 1:** Prevalence of mental health problems in children and young people.

Mental health problem	Prevalence (%) (adapted from Polanczyk et al [3])
Anxiety	6.5
Disruptive behavior	5.7
Oppositional defiance disorder	3.6
Attention-deficit hyperactivity	3.4
Depression	2.6
Conduct	2.1

### Benefits of Digital Health Interventions

Addressing MH problems in CYP is a major public health concern [4,5], which has been impaired by low levels of youth help-seeking behavior [6]. Concerns about stigma and confidentiality, shame or embarrassment in discussing personal issues, financial costs, and/or limited access to services are among the many barriers to accessing help in this population [6-8]. In many instances, existing efficacious face-to-face interventions are adapted using digital technology as a means of addressing these barriers [9]. Digital health interventions (DHIs; eg, internet programs, apps, virtual reality environments, robotic systems) have the potential to be effective, with advantages of accessibility, anonymity, prompt feedback, cost-effectiveness, applicability in real-life contexts, and high treatment fidelity [7,10-15]. Therefore, considering the increased digital literacy and internet use among youth [16], DHIs may serve as a new way to increase accessibility to MH interventions in this population [17,18].

### Efficacy of Digital Health Interventions

The World Health Organization (WHO), the United Kingdom’s National Health Service, and the US National Institute of Mental Health have identified MH apps as cost-effective and scalable solutions for addressing the MH treatment gap [19]. The efficacy of web-based therapies is well established in the treatment of several MH problems, including depression, anxiety, and substance misuse among adolescents [20-22]. Web-based treatment programs have also demonstrated comparable efficacy to face-to-face psychotherapy [23,24]. In addition, smartphone-based MH interventions have been shown to be a promising self-management tool for depression [25] by reducing symptoms, similar to face-to-face interventions. Equivalent results were also found for anxiety-focused mobile apps [26]. Recent systematic reviews have shown that interventions based on computerized cognitive behavioral therapy are a promising and acceptable way to reduce anxiety and depression in CYP [17,18]. The findings also support the clinical benefits of DHIs for other symptoms and disorders such as autism spectrum disorders, attention deficit, and behavioral disorders [27].

### Research on Digital Mental Health Interventions for Children and Young People

Despite the growing interest in using mobile apps to deliver interventions, more research evidence is needed to support implementation in children and young people’s mental health (CYPMH) services [27-29]. For instance, the evaluation of DHIs is increasingly discussed in electronic health research. A recent review showed that the majority of registered DHI evaluation trials employ common methods, such as the randomized controlled trial (RCT) study design [30]. There is much debate in the literature on appropriate methods for evaluating the impact of DHIs [31]. For instance, given the speed at which technologies advance, adaptive research designs may be more useful for increasing usability and ability to respond in a timely manner to users’ experiences [32]. Considering the limitations of traditional research designs, new methodological frameworks and research designs have been developed (eg, continuous evaluation of evolving behavioral intervention technologies [33] and microrandomized trials [34]). To develop DHIs that are more useful and thereby more engaging for users, researchers agree that the impact of different functionalities on levels of engagement is important [35]. Engagement with digital behavior change interventions has been defined in the literature as a subjective experience (the user-perceived state of *flow*, characterized by temporal dissociation, focused attention, interest, and enjoyment) or as a behavior (extent of usage of the DHI over time or adherence) [36]. Perski et al [36] proposed an integrated conceptualization of engagement that includes both the extent (eg, amount, frequency, duration, and depth) of usage and the subjective experience of “what it feels” to be engaged (eg, attention, interest, and affect). However, engagement is usually assessed through the evaluation of the user interaction with the DHI, either by user-reported tools (eg, questionnaires, interviews, or think-aloud studies), by automated recordings of use (eg, log-ins, page views), or by recording physiological or psychophysical correlates of DHI interaction [36].

Despite the potential of DHIs, researchers have identified several limitations that influence practicality [37]. The main limitations identified were restricted tailoring to patient needs, challenges with managing comorbidity and acute crisis [38], low patient engagement and high dropout rates [39]. Although efforts have been made to reduce these occurrences with strategies such as gamification, tailoring, and guided self-help, the aforementioned difficulties remain [27,38,39]. In addition, further challenges arise from cautious attitudes of professionals toward DHIs, such as failure to address important aspects of the disease, data security, and accessibility [40].

### Rationale for This Review

The rapid advancement of technology [41] and the increasing interest of CYP in technology [16] calls for a better understanding and evaluation of DHIs used to *engage* CYP with MH problems [42,43]. Engagement is commonly referred to as the active involvement of participants with the intervention, also described in previous literature as *participation*, *adherence*, *noncompliance*, or *resistance* [44]. This knowledge is crucial to support the development and evaluation of DHIs that are acceptable and feasible in CYPMH settings. This review sets out to contribute to the growing body of knowledge on digital CYPMH interventions by investigating modes of delivery used in DHIs. Although recent meta-analyses highlighted the potential effectiveness of CYPMH DHIs [17,18,45], this review aimed to present information that might be of use in the development of real-world interventions that are more likely to increase engagement from CYP.

### Aims

The primary aim of this study was to review the literature to identify modes of delivery used to engage CYP in digital MH interventions. Second, we explored barriers and facilitators for the usage and implementation of DHIs. The authors also aimed to investigate the ways in which these interventions have been evaluated and whether CYP engage in DHI research. The following questions were addressed:

1. What modes of delivery are used for engaging CYP in digital MH interventions?
2. What are the barriers and facilitators to engaging CYP in digital MH interventions?
3. How do retention rates vary in CYP digital MH intervention research?

## Methods

### Literature Search and Search Strategy

A literature search was conducted using the Cochrane Library, EMBASE, MEDLINE, and PsycINFO databases. All searches were carried out on the same day (December 27, 2018) to control for daily updates. Overall, 3 key concepts informed the search strategy: *child and adolescent mental health*, *digital intervention*, and *engagement*. Terms within similar categories were combined with *OR* and then the results from each category were combined with *AND* (see Multimedia Appendix 1). The search strategy was guided by similar reviews exploring technology or engagement in child and family MH treatment [44-46], the review team discussions, and input from the University College London Institute of Child Health librarian. Reference lists of relevant articles were also scanned for additional potential studies. An initial sample of articles identified through database searching was screened first by titles and abstracts. Next, the full-text versions of potentially relevant studies were retrieved and examined in detail for eligibility at the review team meetings. Differences regarding study selection were resolved by discussion among the authors.

### Inclusion and Exclusion Criteria

Screened articles were included if (1) the study targeted a CYP sample with a mean age less than 25 years; (2) the article described a DHI targeting an MH symptom (related to a primary physical/somatic condition) or the intervention was being used by CYP with MH problems; and (3) the study explored the development or testing of a DHI resulting in data on adherence, acceptability, or barriers and facilitators to engagement. Any study design was deemed acceptable for inclusion. Articles were excluded if (1) the age of the participants was not defined or if the mean age of the sample was 25 years and above, (2) the intervention was for the sole purpose of communicating between a health care professional and the CYP (eg, Skype, email, teleconference, or messages for appointment reminders), (3) the outcome of the study was not clearly defined or did not provide sufficient details to determine if the outcome was directly related to the intervention, and (4) the study had no human participants (eg, discussion articles describing a novel intervention).

### Study Selection Process

**Figure 1:**
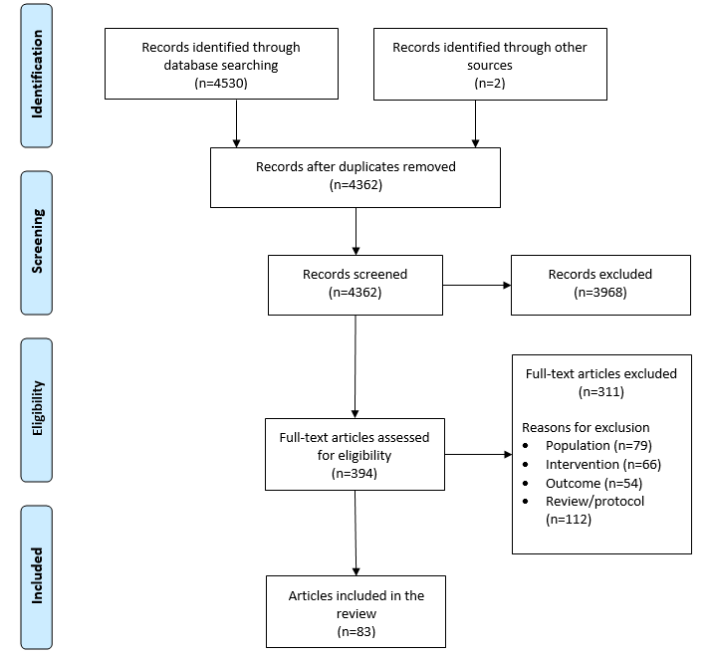
Preferred Reporting Items for Systematic Reviews and Meta-Analyses flow chart of the study selection process.

In accordance with the Preferred Reporting Items for Systematic Reviews and Meta-Analyses guidelines [47], the flowchart presented in Figure 1 provides step-by-step details of our study selection process. After duplicates were removed, at least two members of the review team independently screened the titles and abstracts against the inclusion criteria. The full-text versions of the remaining potential articles were further examined by at least two reviewers for final inclusion. Articles excluded at this stage described interventions that were being used for communication purposes only, did not provide sufficient details of the intervention, were reviews or study protocols, were those in which the age range was not specified or was above the cutoff, targeted a non-MH condition only or targeted parents or clinicians, or had an outcome that was not related to the intervention. Any disagreements were resolved through discussions.

### Data Extraction and Quality Assessment

A standardized form [48] that was adapted and piloted by the review team was used to extract relevant information from each article, including the following: reference, year, country, study aims, study design, sample size, setting, clinical characteristics, type of support including therapeutic treatment, retention rate, outcome, and descriptive characteristics of the sample and the intervention. Study-specific data for the second review question were also extracted at this stage to inform the thematic framework [49]. The mixed methods appraisal tool (MMAT-v2018) [50] was used to assess the methodological quality of each selected study. This tool was discussed in detail and selected based on its ability to report on the quality of varying study designs. Responses were rated on a categorical scale as “no,” “can’t tell,” or “yes” to any of the methodological quality criteria. The number of items rated “yes” was counted to provide an overall score out of a possible 5 [51]. If at least one of the MMAT quality criteria was met, the methodological quality of the study was considered acceptable and the record was included. SL and a second member of the review team independently extracted all data and independently conducted the quality assessment. The 2 reviewers discussed any discrepancies and, if necessary, consulted a third team member to reach a final decision.

### Data Aggregation and Synthesis

The extracted data were collated and summarized to produce a narrative summary of the study characteristics that addressed the first review question. A descriptive numerical summary was presented to group articles by the primary digital platform used to deliver the intervention. SL completed a qualitative data-driven thematic analysis [52] in addition to inductive analysis informed by the Digital Behaviour Change Framework [53] to address the second review question. Moreover, this framework and the capability, opportunity, motivation, and behavior (COM-B) model [54] were used to explore the factors influencing behavior change and intervention design. The coding process involved moving backward and forward between the data and emerging concepts. The first step generated initial codes from open coding, in which units of meanings were derived from a line-by-line analysis followed by axial coding to integrate and differentiate among subcategories. Qualitative findings relating to barriers and facilitators were coded in NVivo [55]. The review team reviewed the coding process, and any disagreements were discussed before reaching a consensus. There were no major disagreements, and consequently, the codes were developed into themes. For the purpose of addressing the third review question, the retention rate was defined as the percentage of participants completing outcome measures for at least one follow-up time point. In studies where this was not explicitly mentioned, we used the percentage of participants continuing to engage with the intervention after a specified period (ie, a time period identified by the original author).

### Changes to the Protocol

Initially, the review team planned to investigate recruitment rates. However, the identified studies varied in recruitment strategies and did not provide sufficient details to address this research question. In addition, although the review team acknowledged the potential of gray literature (eg, research not published in peer-reviewed journals) to broaden the scope of systematic reviews, the team agreed to only include articles published in peer-reviewed journals. This decision was because of various reasons: (1) the popularity of technological advancements in health care; (2) the resource constraints of this study; (3) some evidence of the limited contribution of unpublished studies to the results of meta-analyses in child-relevant reviews [56]; and (4) the consideration that the aim of this systematic review was not related to efficacy and safety, which could be amenable to publication bias. No other substantial deviations from the registered protocol were made. The review protocol was registered in the International Register of Systematic Reviews (PROSPERO) [CRD42018094815].

## Results

### Overview of the Included Articles

The results of this systematic review are presented as a narrative synthesis [57] and, where applicable, descriptive numerical summaries are provided. A total of 83 articles published between 2001 and 2018 met the inclusion criteria (Multimedia Appendix 2) identifying 71 interventions (Multimedia Appendix 3). Of the 83 articles reviewed, almost two-third were conducted in the United States and Canada or in Australia and New Zealand. The most common type of intervention incorporated cognitive behavioral therapy as the main therapeutic modality. The mean age of the included samples was between 2 years to 24 years. Affective disorders (ie, anxiety and depression, including suicidality) were the most common presentation targets of the DHIs reviewed. Multimedia Appendix 4 provides details of the reviewed articles and Table 2 provides a summary of these findings.

A broad range of recruitment strategies were used to develop and test these DHIs, including referrals from health or school professionals; self-referrals through social media and web-based advertising; university email lists; recruitment software; or in-person advertising through posters, flyers, newspaper advertisements, word of mouth, and existing research and support groups. The following section presents the modes of delivery for DHIs, highlighting how they have been evaluated and their main features and purpose. Table 3 provides a summary of the 6 DHI categories identified in this review, the corresponding features, and the study designs adopted.

**Table 2:** Characteristics of the included articles (N=83).

Characteristics			Values, n (%)
**Country**			
	United States and Canada	31 (37)	
	Australia and New Zealand	23 (28)	
	Europe	21 (25)	
	Asia	7 (8)	
	Brazil	1 (1)	
**Therapeutic modality**			
	Cognitive behavioral therapy	39 (47)	
	Cognitive skills training mechanisms	9 (11)	
	Social skills training or social support	7 (8)	
	Applied behavior analysis concepts	3 (4)	
	Single component or combinations^a^	25 (30)	
**Disorders**			
	Affective^b^	38 (46)	
	Attention deficit and hyperactivity	7 (8)	
	Autism spectrum	12 (15)	
	Eating disorders	4 (5)	
	Behavioral disorders^c^	10 (12)	
	Nonspecific or multiple disorders	12 (15)	

^a^Combinations of the following strategies: therapeutic support embedded in positive psychology, behavior activation, self-regulation, learning theories, motivational interviewing, and mindfulness.

^b^Depression, anxiety, or suicidality.

^c^Obsessive compulsive disorder, substance abuse, selective mutism, social difficulties, and psychosis or schizophrenia.

**Table 3:** Summary of digital modes of delivery used in children and young people’s mental health intervention.

Mode of delivery (number of articles)	Goals: features	Study design, n
Website interventions (n=43)	Communication: emails, text messages, social networking, web-based message boards, discussion forums Dissemination of information: text and multimedia channels (videos, animations, and audio), games and quizzes, homework tasks, and web-based profile set up with customizations	RCT^a^ (n=22), pre- to posttest (n=11), observational study—qualitative, quantitative, or mixed methods approaches (n=10)
Games or computer-assisted interventions (n=23)	Dissemination of information, skills development, psychoeducation: photos, stories, animations, quizzes, text messages, and multimedia (audio and videos)	RCT (n=11), pre- to posttest (n=8), observational study—quantitative or mixed methods approaches (n=4)
Apps: web or mobile (n=10)	Dissemination of information, skills development, peer-to-peer communication: text message reminders, text, photos and multimedia (audio and videos) plus an opportunity to upload content	RCT (n=4), pre- to posttest (n=1), observational study—qualitative, quantitative, or mixed methods approaches (n=5)
Robots and digital devices (n=3)	Dissemination of information*:* audio and movement Peer-to-peer communication: email reminders	RCT (n=1), feasibility study (n=1), mixed methods design (n=1)
Virtual reality experiences (n=3)	Dissemination of information, skills development, therapeutic support: Gamification using multimedia (audio and images)	Pre- to posttest (n=2), posttest (n=1)
Mobile text messages (n=1)	Dissemination of information, skills development, social support: Text	RCT (n=1)

^a^RCT: randomized controlled trial.

### What Modes of Delivery Are Used for Engaging Children and Young People in Digital Mental Health Interventions?

#### Website Interventions

Overall, 33 of the 43 articles adopted an interventional study design (22 RCTs and 11 pre- to poststudy designs). The remaining 10 studies adopted observational study designs utilizing qualitative, quantitative, or mixed methods approaches. The methodological quality of the included studies was acceptable. Qualitative studies scores ranged from 2 to 5 points, RCTs and nonrandomized quantitative studies also ranged 2 to 5 points, and mixed methods studies ranged 2 to 4 points.

The primary goal of the majority of the interventions (n=40) was to transmit specific MH information to a targeted population. In addition, 12 of the 40 articles described interventions that were multipurpose, providing an additional opportunity for peer communication (n=7) or for personal health tracking (n=4). YouthCHAT (see Multimedia Appendix 3 for definitions and descriptions of the interventions) provided general information in addition to providing an opportunity for personal health tracking. However, SharpTalk’s primary focus was to facilitate peer-to-peer communication through discussion forums, and Manage Your Life Online functioned as a communication aid that provided an opportunity for personal health tracking.

Various features were adopted to achieve the above goals. Communication occurred digitally using emails, text messages, social networking, web-based message boards, and discussion forums. Dissemination of information occurred through text and multimedia channels (eg, videos, animations, and audio). Some interventions also utilized games and quizzes, homework tasks, and a web-based profile set up with customizations.

#### Games or Computer-Assisted Interventions

Overall, 20 of the 23 articles adopted an interventional study design (11 RCTs and 8 pre- to poststudy designs). The remaining 4 studies adopted observational study designs utilizing quantitative or mixed method approaches. The methodological quality of the included studies varied. RCTs scores ranged 2 to 5 points, nonrandomized quantitative studies ranged 2 to 4 points, and mixed methods ranged 3 to 5 points. No articles used qualitative methods only.

The primary goal of the majority of the interventions (n=18) was to transmit specific MH information to a targeted population. Of the 18 interventions, 4 were multipurpose, providing additional general information to the public (n=1) or an opportunity for personal health tracking (n=4). In addition, 8 interventions focused on cognitive training tasks. The computer-assisted instruction intervention was used as a facilitator to assist children in developing reading skills. The social stories accessed via tablets were also used for psychoeducational purposes.

The gamification approach used to achieve the above goals was accessed either on the web or offline and incorporated photos, stories, animations, quizzes, text messages, and videos.

#### Apps

Of the 10 articles, 5 adopted an interventional study design (4 RCTs and 1 pre- to poststudy design). The remaining 5 articles adopted observational study designs utilizing qualitative, quantitative, or mixed methods approaches. The methodological quality of the included studies varied. Qualitative studies scored either 4 or 5 points, RCTs scores ranged 1 to 4 points, the 1 nonrandomized quantitative study scored 4 points, whereas the 2 mixed methods studies scored 3 points.

The primary goal of most apps was to transmit specific MH information to a targeted population. Furthermore, 3 apps were multipurpose, providing an additional opportunity for personal health tracking. The TECH app further included peer-to-peer communication.

The included apps were either web apps or mobile apps and included text message reminders, text, photos, and multimedia (videos and audio). Users also had the opportunity to upload content such as videos and photos.

#### Robots and Digital Devices

Of the 3 studies, 2 adopted an interventional study design, of which one (CommU) was an RCT. The study on the Fitbit Flex and Facebook adopted a mixed methods design. The study on ARIA adopted an observational study design as a pilot usability study. The methodological quality of the included studies was acceptable. The ARIA study scored 3 points, CommU scored 3 points, and the Fitbit Flex and Facebook intervention study scored 5 points.

The primary goal of ARIA and CommU was to transmit specific MH information to a targeted population, whereas the Fitbit Flex and Facebook intervention additionally provided an opportunity for peer communication.

ARIA and CommU utilized audio and movement to achieve the above purpose. The Fitbit Flex and Facebook synced with 2 other approaches, an app and a website, and included email reminders to achieve its purpose.

#### Virtual Reality Experiences

Furthermore, of the 3 studies, 2 interventions adopted pre- to posttest designs, whereas one adopted a posttest-only design (Virtual Dolphin Interaction). No RCTs were found to evaluate the identified interventions. Cave Automatic Virtual Environment also incorporated a mixed methods approach and obtained qualitative data. The methodological quality was acceptable. The Cave Automatic Virtual Environment and Collaborative Virtual Environment studies scored 3 points and the Virtual Dolphin Interaction study scored 4 points.

The primary goal of all 3 interventions was to transmit specific MH information to a targeted group or to facilitate skills training or provide therapeutic support. Collaborative Virtual Environment also functioned as a communication aid to facilitate collaboration within the virtual reality environment.

Cave Automatic Virtual Environment, Collaborative Virtual Environment, and Virtual Dolphin Interaction utilized features of the gamification approach to engage CYP in a more real-life experience, allowing for more immersion and movement.

#### Mobile Text Messages

In addition, 1 text messaging intervention was identified and evaluated in an RCT. The methodological quality score was 3. The Educating and Supporting Inquisitive Youth in Recovery program aimed to transmit specific MH information to a targeted audience, to facilitate skills training or offer therapeutic support, to provide the opportunity for personal health tracking, and to signpost CYP to additional social support websites. No additional features, apart from text, were described in the study. However, participants were contacted via phone as part of the study.

### What Are the Barriers and Facilitators to Engaging Children and Young People in Digital Mental Health Interventions?

**Figure 2:**
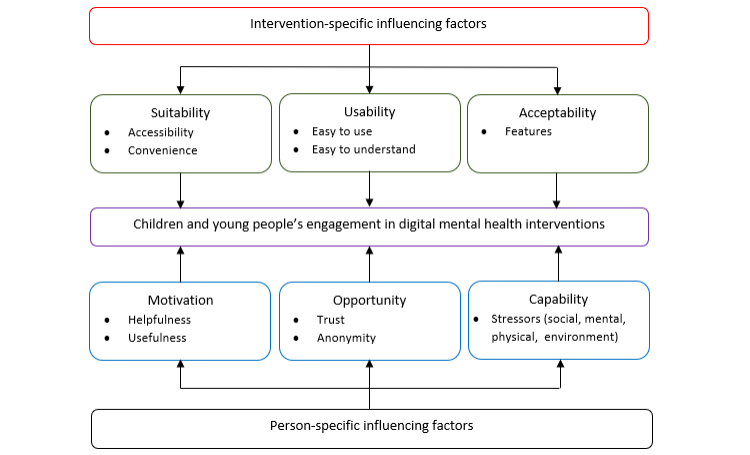
A framework of factors influencing engagement in children and young people’s mental health digital intervention.

Influencing factors presented as barriers and facilitators to engagement emerged as 2 broad themes encompassing 6 factors: intervention-specific influences (suitability, usability, and acceptability) and person-specific influences (motivation, capability, and opportunity). Overall, 29% (24/83) of the included articles provided data to support these themes, 13 studies provided data for suitability, 13 for usability, and 14 provided data for acceptability. Of the 24 articles, 8 provided data to inform motivation, 4 for capability, and 13 for opportunity. A summary of concepts corresponding to the individual factors within the major themes is presented in Multimedia Appendix 5. On the basis of these findings, a conceptual framework (Figure 2) was developed, highlighting the specific components impacting engagement in CYP digital MH interventions, which can inform the development of, and research into, CYP DHIs.

#### Theme 1: Intervention-Specific Influences

CYP highlighted factors related to the development of the intervention, which influenced whether they used the intervention or not. A prominent factor influencing the acceptability (ie, willingness to use) of the intervention was the features, whereby CYP highlighted certain images, specific language, and interfaces that were unappealing to them. They made suggestions and highlighted features such as videos, having less text, ability to personalize or create a profile, and ability to connect with others or receive text message reminders as encouraging their use of the intervention. CYP also suggested that providing rewards could also be a motivating factor for engaging with DHIs. Similarly, usability (ie, the degree to which the DHI was able to be used) was important for promoting engagement. Interventions that CYP favored were described as self-paced, user friendly, age appropriate, simple, and straightforward. However, in situations where CYP had problems understanding the task, or if the intervention did not provide sufficient instructions on usage, they were less inclined to continue using the DHI.

Another main factor was the suitability (ie, the degree to which the DHI is in line with daily activities) of the intervention to the lifestyle of CYP. Although CYP liked not having to travel to access the intervention and the ability to use it while at home, they were *put off* by technical issues or having to use media such as emails or desktop computers that they used less frequently in their daily lives. Many CYP highlighted not participating in the intervention because of a lack of time and inability to integrate the task into their everyday life. They suggested that flexibility concerning time and ability to bypass long waiting lists encouraged usage. In addition, they highlighted that DHIs were convenient and welcomed as they spend most of their time on the web.

#### Theme 2: Person-Specific Influences

Of the 6 factors, 3 were associated with person-specific barriers and facilitators to behavior change, which is in line with the COM-B model [54]. The opportunity for the intervention to be adopted was highlighted in 3 areas. First, feeling a sense of connectedness was important to CYP. They were more likely to use the intervention if it facilitated conversations with others because they wanted to know that others had similar experiences. Some CYP even indicated that they “felt alone” on the web without the support of a therapist. Trust was also of great importance to CYP, and they were reluctant to accept DHIs because of privacy concerns or uncertainties around its validity. CYP made suggestions to use trusted *brand names* that they were familiar with. They were also more inclined to use the interventions if there was transparency or evidence provided to support its credibility. The concerns around trust also extended to their preference for anonymity. They highlighted that anonymity made it easier to talk to a stranger on the web without feelings of embarrassment.

The second major factor identified as a person-specific influence was motivation. Some tasks were of less interest to CYP, and sometimes, they would have forgotten the existence of the intervention. However, they highlighted that curiosity and perceived need influenced their usage. Perceived usefulness of the intervention to address their needs was a major motivating factor; therefore, if the resource was viewed as unhelpful or too general, CYP were less interested in using it. However, although CYP were eager to use DHIs, the capability to engage with the intervention was sometimes affected by physical, environmental, and MH stressors, representing another major influencing factor.

### How Do Retention Rates Vary in Children and Young People Digital Mental Health Intervention Research?

Owing to the heterogeneity in study design and intervention type, not all articles provided sufficient detail to estimate retention rates. Therefore, we were unable to include estimates for 16 of the included articles. The average retention rate across the remaining 67 articles showed almost 80% of CYP using DHIs or completing the follow-up measures. Results showed that the retention rates varied, with 11.9% (8/67) of articles achieving 100% retention and 8.95% (6/67) reporting less than 50% retention. Overall, 83.58% (56/67) of the included articles had a retention rate of at least 70%. Subgroup analyses for approaches with a larger number of articles indicated that the average retention rate for games and computer-assisted intervention studies was 86.95%, followed by websites interventions with 78.87%, and apps with 78.45%. Multimedia Appendix 4 shows the distribution of retention rates across studies.

## Discussion

### Overview

This review identified 6 modes of delivery of DHIs for CYP with MH issues: websites, games and computer-assisted programs, apps, robots and digital devices, virtual reality, and mobile text messaging. Overall, 2 themes emerged, highlighting intervention-specific and person-specific barriers and facilitators to engagement in DHIs. In addition, the findings of this review suggest a high average retention rate of almost 80% when the identified modalities were evaluated. Knowledge of these approaches, including influencing factors to usage from the perspective of the CYP, provided information that can be used to design and evaluate new DHIs.

### Explanation of the Overall Findings

From the articles reviewed, 59 contributions were published between 2013 and 2018, with 15 records published in 2018. This is consistent with the fact that interest in applying digital technologies to MH practice has been increasing since the early 2000s, and recommendations for research in this field were issued only in 2013 [58,59]. With most of the studies reviewed being conducted in developed countries, digital responses to CYPMH seem unbalanced. Previous research [60-62] highlighted the paucity of access to DHIs in low- and middle-income countries. This inequality could be because of limited resources (both financial and human), shortage of skilled personnel, infrastructure problems leading to poor internet penetration and connectivity [63], or the absence of a specific CYPMH policy [64]. However, a strong association between the severity of risk for mood disorders and social disadvantage has been documented [65,66]. Anxiety is the most common psychiatric condition affecting CYP in all societies [45,67-69]. It may also co-occur with other disorders, both concurrently and sequentially, leading to further health problems [68]. Therefore, it is not surprising, that 46% of articles resulting from our literature search targeted CYP with affective disorders.

The modes of delivery identified in this review are similar to those identified in other reviews exploring adolescent physical health [70,71] and MH [45]. The purpose of the interventions is also in line with WHO’s classification of DHIs [72]. The intervention-specific and person-specific influences on CYP engagement identified in the present review mirror those of previous research on the broader technology acceptance model [73-75], the conceptual framework for engagement in Digital Behavior Change Interventions in adults [36,53], and more recently, the application of social cognitive theory to understand engagement with DHIs for trauma recovery [35]. For these models, predictors included perceived need, engagement self-efficacy, outcome expectations, and symptom severity. The proposed model of CYP engagement in digital MH interventions, based on the findings of the present review, builds on these models by highlighting the importance of the social context in which young people engage with DHIs. It also highlights major factors for researchers and developers in CYPMH to facilitate opportunities for a sense of connectedness. *Peer -connectedness* may be challenged by the necessary application of safeguarding measures. Indirect peer connectedness where CYP can access appropriately anonymous and asynchronous stories from real CYP with similar experiences may be one such approach, as in SharpTalk [76]. Similarly, options for anonymous usage may be particularly important for CYP’s *self-connectedness* in terms of sharing experience in a manner that mitigates the role of stigma and shame [77,78]. *Professional connectedness* was particularly important in DHIs as CYP wanted to connect with a trusted support provider in lieu of connecting with a professional in real life. A key facilitator of professional connectedness was credibility in relation to evidence of the intervention’s effectiveness and trust in the privacy and data security, which could be facilitated by using familiar *brand names*. Although characterized as an intervention-specific factor in other models [53], we characterized this as part of opportunities for connectedness and, therefore, as a person-specific factor. Future studies should explore the impact of new modes of delivery to promote a sense of connectedness in DHIs (eg, more usage of features such as ChatBots, as in the Manage Your Life Online intervention).

Other barriers and facilitators that were identified in this review also emphasize the importance of user-centered design methods when developing DHIs for CYP [53]. Through co-design workshops and focus groups with CYP, developers can ensure that a DHI’s design is age appropriate, (eg, little text and using youth-engaging language) by putting a greater focus on videos and pictures, while keeping the platform user friendly. Moreover, CYP mentioned factors such as reward systems and reminders, which fall under the umbrella of persuasive design methods and have been explored in previous research [36,79]. The positive influence of these methods on user engagement and adherence to DHIs has been supported; however, quantifiable evidence from trials is still lacking [36,79]. With respect to reminders, past research has indicated a positive impact on engagement. However, excessive and undue reminders have also been shown to have opposing effects [80]. Evidence from previous studies has suggested that specific behavior change techniques, such as goal setting or self-monitoring tools, relate to higher engagement [81]. This review did not extensively investigate these techniques and therefore cannot fully suggest their potential positive effects on the engagement of CYP. However, the findings of this review justify that designing DHIs with CYP in mind would be ideal to promote usage, adherence, and positive user experience and to address the barriers that some of the reviewed studies suggest.

### Comparison of Research Retention Rates With Other Studies

Our findings suggest that the retention rate of CYP in digital MH interventions (mean retention rate of 79.2%) was superior to that reported in face-to-face CYP MH outpatient care, where dropout affects 20% to 60% of the cases [82]. However, a direct comparison with other studies is not clear, given the diversity of criteria used for defining dropout. For instance, dropout percentages are lower when dropout is defined by the therapist’s opinion than when dropout is defined by the completion of a certain number of sessions [83-85]. Our definition of retention relied on completion of the first follow-up measure or engagement for a specified period, which may explain the higher retention found. In this review, retention rates also varied widely across the studies (range 15.79%-100%). A similar heterogeneity in retention rates was found in previous reviews of studies with adults receiving internet-based MH programs (17%-98%) [82], as well as in face-to-face MH interventions with CYP [83], and the adult population (varying between 17%-72% and 17%-98%, respectively) [86]. Efficacy studies tend to present lower dropout rates than studies conducted in naturalistic settings [83]. Our review included a variety of empirical studies, which may have contributed to the diversity of retention rates found. Finally, the average retention rate for games and computer-assisted intervention studies was almost 10% higher than that of the other modalities, which may reflect the preference of children for interventions in game formats [87,88].

### Digital Mental Health Care and Support of Children and Young People

DHIs were included at various stages of the provision of psychological support. Technology-mediated programs and tools were part of prevention, assessment, treatment (psychoeducation and psychotherapy) and follow-up of MH care. This extensive potential of DHIs can support the WHO’s initiative to identify and intervene to lessen the MH treatment gap [89]. When used as part of the initial assessment, support for shared decision making, personalized goal setting, progression, or management of transitions, DHIs are able to support CYP by enhancing their sense of agency and control. This may in turn promote greater involvement in the treatment process [90]. In several of the reviewed studies, DHIs targeting social skills training and joint attention training were used in the initial phase as a facilitator of the therapeutic process. For some specific conditions such as social anxiety, selective mutism, autism spectrum disorders, and attention-deficit conditions, the involvement of digitized programs in the preliminary phase of therapy may be an important facilitator for therapeutic success [91,92]. DHIs as part of the therapeutic process can be used in the periods between face-to-face treatment for interactive homework assignments, reminders, self-monitoring tools, individualized exercises, and real-time symptom assessment.

### Implications and Recommendations

DHIs can be a helpful way to support and treat MH problems. Such tools can complement the various stages of the provision of psychological support or psychotherapy among CYP with MH problems. However, effective implementation and sustained usage will rely on the extent to which the design is appropriate for the intended purpose and how it will be used in practice. This understanding may help to minimize the risks associated with fear of usage that some end users experience by providing useful directions on how to design technologically responsible therapeutic approaches [93]. As a result, the findings of this review suggest that the development of DHIs should be suitable for CYP’s lifestyle, focusing on ease of access, such as the ability to be used on their mobile devices at their convenience. Attention should also be given to the design of DHIs to ensure that it is not too complex and that the features are attractive to CYP. In addition, incorporating concepts that provide the CYP with a level of trust for the DHI and the ability to connect with others should be carefully considered. To target concerns about usefulness, developers should work with clinicians and CYP to ensure that suitable information is provided through the intervention. This collaborative approach can highlight specific ways to encourage CYP to continue engaging or increasing engagement during stressful periods.

Although this study provides insight that is valuable for the development of new interventions, future research should (1) not only focus on the effectiveness but also investigate engagement, taking into account influencing factors, as an important component of research; (2) arrive at a consensus on defining engagement and how it should be measured; (3) provide adequate reporting of recruitment and retention rates; and (4) compare CYP preferences for various modes of delivery or therapeutic approaches. Finally, this study also acknowledges the implications for practice. The findings suggest CYP interest in DHIs, and therefore, (1) efforts to improve engagement may be beneficial to CYPMH outcomes and (2) families including the CYP and clinicians should work together to identify DHIs that are suitable to the CYP’s lifestyle.

### Strengths and Limitations

This review adhered to established guidelines for systematic reviews [94] and adopted a comprehensive study design carried out by a team of researchers, allowing each stage of the review to be undertaken by at least two independent reviewers. Most importantly, this review highlighted the range of modes of delivery, factors influencing usage, and the variation in study types and retention of CYP in digital MH intervention studies. Our findings contribute to a broader understanding of the CYP DHI literature. However, this review is not devoid of limitations. The review team attempted to identify and include as many articles as possible; however, unknowingly and unintentionally, some papers may have been missed. This can be partly because of the challenges and inconsistencies when defining the construct of engagement resulting in a wide variety of terms used [44]. In addition, unpublished data were not included in the search strategy, which may have impacted the results of this review. Nevertheless, this approach was also seen as a further strength by ensuring that only peer-reviewed interventions were included. Moreover, the study team attempted to group interventions based on digital platforms to describe each approach. However, there may still be some variation within these groupings that make it difficult to categorize. This review was also limited as only subsamples of the total number of included articles contributed to addressing research questions 2 and 3. Therefore, caution was taken when generalizing the findings and drawing overall conclusions. In addition, because of the variability in study designs, we were prudent when averaging retention rates for this review, as follow-up measures were collected at varying time points across the selected records.

### Conclusions

DHIs may be of interest to CYP, particularly in the area of MH treatment. Research on retention rates suggests high engagement of CYP in digital MH interventions that may encourage further development of DHIs in the near future. CYPMH services could benefit from this development as the included studies indicate. However, the results of this review highlighted intervention-specific and person-specific factors that influence CYP usage of digital MH interventions that should be considered. With continuous technological advancements, it is desirable to know which modalities may increase usability and adherence to better support CYP facing MH challenges.

## Appendix

Multimedia Appendix 1 Search strategy.

Multimedia Appendix 2 Reviewed articles.

Multimedia Appendix 3 Intervention characteristics.

Multimedia Appendix 4 Characteristics of reviewed articles.

Multimedia Appendix 5 Themes and supporting codes.

## Abbreviations

COM-B: capability, opportunity, motivation, and behavior
CYP: children and young people
CYPMH: children and young people’s mental health
DHI: digital health intervention
MH: mental health
MMAT: mixed methods appraisal tool
RCT: randomized controlled trial
WHO: World Health Organization
