# Supplementary material for: Engaging Children and Young People in Digital Mental Health Interventions: Systematic Review of Modes of Delivery, Facilitators, and Barriers
Source: J Med Internet Res. 2020 Jun 23;22(6):e16317. doi: 10.2196/16317 (PMC7381028; doi:10.2196/16317)
Supplement: Multimedia Appendix 1 [file jmir_v22i6e16317_app1.pdf]

Databases, search terms, and search results

PsycINFO

27.12.18; **1565**

.sh=subject heading; .mp=title, abstract, heading word, table of contents,

key concepts, original title, tests & measures; .ti=title; .ab=abstract; .md=methodology; .id=key concepts (IV, DV, population)

Child & Adolescent Mental Health	Digital Intervention	Engagement
(child* OR youth* OR young OR adolesce* OR teenage*).mp. AND (exp child psychiatry/ OR exp adolescent psychiatry/ OR exp child psychopathology/ OR exp adolescent psychopathology/ OR exp mental health/ OR exp community mental health service/ OR exp community psychiatry/ OR exp emotional adjustment/ OR exp anxiety disorder/ OR exp agoraphobia/ OR exp mental health programs/ OR exp mental health services/ OR exp primary mental	(exp computer assisted therapy/ OR exp online therapy/ OR exp internet/ OR exp computer applications/ OR exp mobile devices/ OR exp social media/ OR exp websites/ OR exp internet usage/).sh. OR (app* OR mobile* OR digital* OR technolog* OR computer* OR mhealth* OR mobile health OR ehealth OR electronic health OR social media OR	(exp involvement/ OR exp client participation/ OR exp participation/ OR exp treatment compliance/ OR exp cooperation/ OR exp resistance/ OR exp psychotherapy resistance/).sh. OR (engag* OR participat* OR involv* OR adherence OR noncompliance* OR compliance* acceptab* OR resistance*).ti.

health prevention/ OR exp well being/ OR exp abnormal psychology/ OR exp attention deficit disorder/ OR exp attention deficit disorder with hyperactivity/ OR exp behavior disorders/ OR exp communication disorders/ OR exp conduct disorder/ OR exp emotional adjustment/ OR exp emotional disturbances/ OR exp intellectual development disorder/ OR exp learning disorder/ OR exp psychiatric patients/ OR exp psychopathology/ OR exp suicide/ OR exp thought disturbances/ OR exp psychotherapeutic outcomes/ OR exp mental health program evaluation/).sh.	phone OR online OR web-based OR internet OR chatbot).ti,ab.	

MEDLINE

27.12.18; **858**

EN

Kf=keyword heading word

Child & Adolescent Mental Health	Digital Intervention	Engagement
(Child/ or Adolescent/).sh or child* OR youth* OR young OR adolesce* OR teen* or student* or girl* or boy* or pupil*) .tw AND (Mental Health/ or Depression/ or Emotions/ or Stress, Psychological/ or Mental Health Services/ or Community Mental Health Services/ or Psychotic Disorders/ or Mental Disorders/ or Community Mental Health Centers/ or Mental Health Services/).sh OR ("mental difficult*" or "mental health challenge*" or "mental health problem" or "mental challenge" or "mental well$being" or "mental health well$being").tw	(Therapy, Computer-Assisted/ or Internet/ or Computers/ or Diagnosis, Computer-Assisted/ or Computer Communication Networks/ or Mobile Applications/).sh OR (email* or app* or mobile* or e-mail* or digital* or technolog* or computer* or tablet* or mhealth* or mobile health or ehealth or electronic health or social media or phone or website* or online or web-based or internet or chatroom* or chatbot or laptop).tw	(exp Patient Participation/px [Psychology] or Patient Compliance/px [Psychology] or Cooperative Behavior/).sh OR (involv* or "Treatment Compliance" or COOPERATION or RESISTANCE or "PSYCHOTHERAPEUTIC RESISTANCE" or engag* or participat* or involv* or adherence or noncompliance* or compliance* cooperat* or resistance*).ti.

Cochrane

27.12.18; **1109**

Child & Adolescent Mental Health	Digital Intervention	Engagement
exp Child Psychiatry or exp Adolescent Psychiatry or exp Mental Health or exp Community Mental Health Services or exp Community Psychiatry or exp Emotional Adjustment or exp Anxiety Disorders or exp Agoraphobia or exp Mental Health Services or exp Child Welfare or exp Attention Deficit Disorder with Hyperactivity or exp Mental Disorders or exp or exp Communication Disorders or exp Conduct Disorder or exp Emotional Adjustment or exp Affective Symptoms or exp Intellectual Disability or exp	(app* or mobile device or mobile app or smartphone or mobile phone or digital* or technolog* or computer* or mhealth* or m-health or mobile health or ehealth or electronic health or social media or phone or online or web-based) or exp Therapy, Computer-Assisted or exp Internet or exp Software or exp Social Media or internet AND prevention or treatment* or intervention* or therap* or training or support* or help* or assistan*	(engag* or participat* or involv* or adherence or noncompliance* or compliance* cooperat* or resistance*) or exp Patient Participation or exp Patient Compliance or Cooperative Behavior

Learning Disorders or exp Psychopathology or exp Suicide OR ““child psychiatry” or “adolescent psychiatry” or “child psychopathology” or “adolescent psychopathology” or “mental health” or “community mental health service*” or “community psychiatry” or “emotional adjustment” or “anxiety disorder*” or agoraphobia or “mental health program*” or “mental health service*” or “primary mental health prevention” or “well being” or “abnormal psychology” or “attention deficit disorder*” or “attention deficit disorder with hyperactivity” or “behavior disorder*” or “communication disorder*” or “conduct disorder” or “emotional adjustment” or “emotional disturbance*” or “intellectual development disorder*” or “learning disorder*” or “psychiatric patient*” or psychopathology or suicide or “thought		

disturbance*" or "psychotherapeutic outcome*" or "mental health program evaluation" OR (mental difficult* or mental health challenge* or mental health difficult* or mental health problem* or mental challenge* or mental well$being or mental health well$being or mental illness or mental health illness or mood disorder* **AND** child* or teen* or young person* or young people or kid* or adolescen* or infan* or toddler* or young adult* or student* or girl* or boy* or pupil* or exp child or exp adolescent or explode foster child		

EMBASE

27.12.18; **998**

Child & Adolescent Mental Health	Digital Intervention	Engagement
(child* or teen* or "young person*" or "young people" or kid* or adolescen* or infan* or toddler* or "young adult*" or student* or girl* or boy* or pupil*).ti. OR (exp child/ or exp Adopted children/ or exp Foster Children/).sh AND exp child psychiatry/ or exp adolescent psychiatry/ or exp adolescent psychopathology/ or exp mental health/ or exp community mental health service/ or exp community psychiatry/ or exp emotional adjustment/ or exp anxiety disorder/ or exp agoraphobia/ or exp mental health programs/ or exp mental health services/ or exp primary mental health prevention/ or exp well being/ or exp abnormal psychology/ or exp attention deficit	exp computer assisted therapy/ or exp internet/ or exp computer applications/ or exp mobile devices/ or exp social media/ or exp websites/ or exp internet usage/ OR (("online therap*" or app* or "mobile device*" or "mobile app*" or smartphone* or "mobile phone8" or digital* or technolog* or computer* or mhealth* or m-health or mobile health or ehealth or "electronic health" or "social media" or phone or online or web-based) adj2 (treatment* or intervention* or therap* or training or support* or help* or assistan*)).ti.	exp client participation/ or exp participation/ or exp Treatment Compliance/ or exp cooperation/ or exp resistance/ or exp psychotherapeutic resistance/ OR (engag* or participat* or involv* or adherence or noncompliance* or compliance* cooperat* or resistance*).tw.

disorder/ or exp attention deficit disorder with hyperactivity/ or exp behavior disorders/ or exp communication disorders/ or exp conduct disorder/ or exp emotional adjustment/ or exp emotional disturbances/ or exp intellectual development disorder/ or exp learning disorder/ or exp psychiatric patients/ or exp psychopathology/ or exp suicide/ or exp thought disturbances/ or exp psychotherapeutic outcomes/ or exp mental health program evaluation/ or ("child psychopathology" or "mental difficult*" or "mental health challenge*" or "mental health difficult*" or "mental health problem*" or "mental challenge*" or "mental well$being" or "mental health well$being" or "mental illness" or "mental health illness" or "mood disorder*").tw.		
