# Supplementary material for: Engaging Children and Young People in Digital Mental Health Interventions: Systematic Review of Modes of Delivery, Facilitators, and Barriers
Source: J Med Internet Res. 2020 Jun 23;22(6):e16317. doi: 10.2196/16317 (PMC7381028; doi:10.2196/16317)
Supplement: Multimedia Appendix 2 [file jmir_v22i6e16317_app2.docx]

**Reviewed Articles**

1. Wozney L, Baxter P, Newton AS. Usability evaluation with mental health professionals and young people to develop an Internet-based cognitive-behaviour therapy program for adolescents with anxiety disorders. BMC Pediatr. 2015;15:213.

2. Price M, Yuen EK, Davidson TM, Hubel G, Ruggiero KJ. Access and completion of a Web-based treatment in a population-based sample of tornado-affected adolescents. Psychol Serv. 2015;12(3):283-90.

3. March S, Spence SH, Donovan CL. The efficacy of an internet-based cognitive-behavioral therapy intervention for child anxiety disorders. J Pediatr Psychol. 2009;34(5):474-87.

4. Johnston L, Dear BF, Gandy M, Fogliati VJ, Kayrouz R, Sheehan J, Rapee RM, Titov N. Exploring the efficacy and acceptability of Internet-delivered cognitive behavioural therapy for young adults with anxiety and depression: an open trial. Aust N Z J Psychiatry. 2014;48(9):819-27.

5. Fortier MA, Bunzli E, Walthall J, Olshansky E, Saadat H, Santistevan R, Mayes L, Kain ZN. Web-Based Tailored Intervention for Preparation of Parents and Children for Outpatient Surgery (WebTIPS). Anesthesia & Analgesia. 2015;120(4):915-22.

6. Anderson RE, Spence SH, Donovan CL, March S, Prosser S, Kenardy J. Working alliance in online cognitive behavior therapy for anxiety disorders in youth: comparison with clinic delivery and its role in predicting outcome. J Med Internet Res. 2012;14(3):e88.

7. Tillfors M, Andersson G, Ekselius L, Furmark T, Lewenhaupt S, Karlsson A, Carlbring P. A randomized trial of Internet-delivered treatment for social anxiety disorder in high school students. Cogn Behav Ther. 2011;40(2):147-57.

8. Bidargaddi N, Musiat P, Winsall M, Vogl G, Blake V, Quinn S, Orlowski S, Antezana G, Schrader G. Efficacy of a Web-Based Guided Recommendation Service for a Curated List of Readily Available Mental Health and Well-Being Mobile Apps for Young People: Randomized Controlled Trial. Journal of medical Internet research [Internet]. 2017 2017/05//; 19(5):[e141 p.]. Available from: http://europepmc.org/abstract/MED/28500020

http://europepmc.org/articles/PMC5446666

https://doi.org/10.2196/jmir.6775.

9. Silfvernagel K, Gren-Landell M, Emanuelsson M, Carlbring P, Andersson G. Individually tailored internet-based cognitive behavior therapy for adolescents with anxiety disorders: A pilot effectiveness study. Internet Interventions. 2015;2(3):297-302.

10. Schlosser DA, Campellone TR, Truong B, Etter K, Vergani S, Komaiko K, Vinogradov S. Efficacy of PRIME, a Mobile App Intervention Designed to Improve Motivation in Young People With Schizophrenia. Schizophr Bull. 2018;44(5):1010-20.

11. Moir F, Fernando AT, Kumar S, Henning M, Moyes SA, Elley CR. Computer assisted learning for the mind (CALM): The mental health of medical students and their use of a self-help website. New Zealand Medical Journal. 2015;128(1411):51-8.

12. Saekow J, Jones M, Gibbs E, Jacobi C, Fitzsimmons-Craft EE, Wilfley D, Barr Taylor C. StudentBodies-eating disorders: A randomized controlled trial of a coached online intervention for subclinical eating disorders. Internet Interventions. 2015;2(4):419-28.

13. Backman A, Mellblom A, Norman-Claesson E, Keith-Bodros G, Frostvittra M, Bölte S, Hirvikoski T. Internet-delivered psychoeducation for older adolescents and young adults with autism spectrum disorder (SCOPE): An open feasibility study. Research in Autism Spectrum Disorders. 2018;54:51-64.

14. Manicavasagar V, Horswood D, Burckhardt R, Lum A, Hadzi-Pavlovic D, Parker G. Feasibility and effectiveness of a web-based positive psychology program for youth mental health: randomized controlled trial. J Med Internet Res. 2014;16(6):e140.

15. Petsky HL, Newcombe P, Christie T, Casey L, Sheffield J, Anderson-James S, Scrivener G, Chang AB. Breathe Easier Online (BEO): Improving the psychosocial well-being of children with chronic respiratory illnesses. Paediatric Respiratory Reviews. 2010;11:S103-S.

16. Santisteban DA, Czaja SJ, Nair SN, Mena MP, Tulloch AR. Computer Informed and Flexible Family-Based Treatment for Adolescents: A Randomized Clinical Trial for at-Risk Racial/Ethnic Minority Adolescents. Behavior Therapy. 2017;48(4):474-89.

17. Landback J, Prochaska M, Ellis J, Dmochowska K, Kuwabara SA, Gladstone T, Larson J, Stuart S, Gollan J, Bell C, Bradford N, Reinecke M, Fogel J, Van Voorhees BW. From prototype to product: development of a primary care/internet based depression prevention intervention for adolescents (CATCH-IT). Community Ment Health J. 2009;45(5):349-54.

18. Saulsberry A, Marko-Holguin M, Blomeke K, Hinkle C, Fogel J, Gladstone T, Bell C, Reinecke M, Corden M, Van Voorhees BW. Randomized Clinical Trial of a Primary Care Internet-based Intervention to Prevent Adolescent Depression: One-year Outcomes. Journal of the Canadian Academy of Child and Adolescent Psychiatry = Journal de l'Academie canadienne de psychiatrie de l'enfant et de l'adolescent. 2013;22(2):106.

19. Gladstone T, Marko-Holguin M, Henry J, Fogel J, Diehl A, Van Voorhees BW. Understanding Adolescent Response to a Technology-Based Depression Prevention Program. Journal of Clinical Child & Adolescent Psychology. 2013;43(1):102-14.

20. Iloabachie C, Wells C, Goodwin B, Baldwin M, Vanderplough-Booth K, Gladstone T, Murray M, Fogel J, Van Voorhees BW. Adolescent and parent experiences with a primary care/Internet-based depression prevention intervention (CATCH-IT). Gen Hosp Psychiatry. 2011;33(6):543-55.

21. Van Voorhees BW, Fogel J, Pomper BE, Marko M, Reid N, Watson N, Larson J, Bradford N, Fagan B, Zuckerman S, Wiedmann P, Domanico R. Adolescent Dose and Ratings of an Internet-Based Depression Prevention Program: A Randomized Trial of Primary Care Physician Brief Advice versus a Motivational Interview. Journal of cognitive and behavioral psychotherapies : the official journal of the International Institute for the Advanced Studies of Psychotherapy and Applied Mental Health. 2009;9(1):1-19.

22. Van Voorhees BW, Fogel J, Reinecke MA, Gladstone T, Stuart S, Gollan J, Bradford N, Domanico R, Fagan B, Ross R, Larson J, Watson N, Paunesku D, Melkonian S, Kuwabara S, Holper T, Shank N, Saner D, Butler A, Chandler A, Louie T, Weinstein C, Collins S, Baldwin M, Wassel A, Vanderplough-Booth K, Humensky J, Bell C. Randomized clinical trial of an Internet-based depression prevention program for adolescents (Project CATCH-IT) in primary care: 12-week outcomes. J Dev Behav Pediatr. 2009;30(1):23-37.

23. Motter JN, Grinberg A, Lieberman DH, Iqnaibi WB, Sneed JR. Computerized cognitive training in young adults with depressive symptoms: Effects on mood, cognition, and everyday functioning. J Affect Disord. 2019;245:28-37.

24. Spence SH, Holmes JM, March S, Lipp OV. The feasibility and outcome of clinic plus internet delivery of cognitive-behavior therapy for childhood anxiety. J Consult Clin Psychol. 2006;74(3):614-21.

25. Sobowale K, Zhou AN, Van Voorhees BW, Stewart S, Tsang A, Ip P, Fabrizio C, Wong KL, Chim D. Adaptation of an internet-based depression prevention intervention for Chinese adolescents: from "CATCH-IT" to "grasp the opportunity". Int J Adolesc Med Health. 2013;25(2):127-37.

26. Monshat K, Vella-Brodrick D, Burns J, Herrman H. Mental health promotion in the Internet age: a consultation with Australian young people to inform the design of an online mindfulness training programme. Health Promot Int. 2012;27(2):177-86.

27. Ooi YP, Raja M, Sung S, Fung D, Koh J. Application of a web-based cognitive-behavioural therapy programme for the treatment of selective mutism in Singapore: A case series study. Singapore medical journal. 2012;53:446-50.

28. Alvarez-Jimenez M, Gleeson JF, Bendall S, Penn DL, Yung AR, Ryan RM, Eleftheriadis D, D'Alfonso S, Rice S, Miles C, Russon P, Lederman R, Chambers R, Gonzalez-Blanch C, Lim MH, Killackey E, McGorry PD, Nelson B. Enhancing social functioning in young people at Ultra High Risk (UHR) for psychosis: A pilot study of a novel strengths and mindfulness-based online social therapy. Schizophr Res. 2018;202:369-77.

29. Neil AL, Batterham P, Christensen H, Bennett K, Griffiths KM. Predictors of adherence by adolescents to a cognitive behavior therapy website in school and community-based settings. J Med Internet Res. 2009;11(1):e6.

30. Lillevoll KR, Vangberg HCB, Griffiths KM, Waterloo K, Eisemann MR. Uptake and adherence of a self-directed internet-based mental health intervention with tailored e-mail reminders in senior high schools in Norway. BMC psychiatry. 2014;14(1):14.

31. O'Kearney R, Kang K, Christensen H, Griffiths K. A controlled trial of a school-based Internet program for reducing depressive symptoms in adolescent girls. Depress Anxiety. 2009;26(1):65-72.

32. O'Kearney R, Gibson M, Christensen H, Griffiths KM. Effects of a cognitive-behavioural internet program on depression, vulnerability to depression and stigma in adolescent males: a school-based controlled trial. Cogn Behav Ther. 2006;35(1):43-54.

33. Calear AL, Christensen H, Mackinnon A, Griffiths KM. Adherence to the MoodGYM program: outcomes and predictors for an adolescent school-based population. J Affect Disord. 2013;147(1-3):338-44.

34. Gaffney H, Mansell W, Edwards R, Wright J. Manage Your Life Online (MYLO): A Pilot Trial of a Conversational Computer-Based Intervention for Problem Solving in a Student Sample. Behavioural and Cognitive Psychotherapy. 2013;42(6):731-46.

35. Anderson RA, Rees CS, Finlay-Jones AL. Internet-based cognitive-behavioural therapy for young people with obsessive-compulsive disorder: Lessons learned. Journal of Obsessive-Compulsive and Related Disorders. 2017;15:7-12.

36. Sweeney GM, Donovan CL, March S, Forbes Y. Logging into therapy: Adolescent perceptions of online therapies for mental health problems. Internet Interv. 2019;15:93-9.

37. Hetrick SE, Yuen HP, Bailey E, Cox GR, Templer K, Rice SM, Bendall S, Robinson J. Internet-based cognitive behavioural therapy for young people with suicide-related behaviour (Reframe-IT): a randomised controlled trial. Evidence Based Mental Health. 2017;20(3):76.

38. Jones R, Sharkey S, Ford T, Emmens T, Hewis E, Smithson J, Sheaves B, Owens C. Online discussion forums for young people who self-harm: user views. The Psychiatrist. 2018;35(10):364-8.

39. Kass AE, Trockel M, Safer DL, Sinton MM, Cunning D, Rizk MT, Genkin BH, Weisman HL, Bailey JO, Jacobi C, Wilfley DE, Taylor CB. Internet-based preventive intervention for reducing eating disorder risk: A randomized controlled trial comparing guided with unguided self-help. Behav Res Ther. 2014;63:90-8.

40. Jones M, Luce KH, Osborne MI, Taylor K, Cunning D, Doyle AC, Wilfley DE, Taylor CB. Randomized, controlled trial of an internet-facilitated intervention for reducing binge eating and overweight in adolescents. Pediatrics. 2008;121(3):453-62.

41. Fichter MM, Quadflieg N, Nisslmuller K, Lindner S, Osen B, Huber T, Wunsch-Leiteritz W. Does internet-based prevention reduce the risk of relapse for anorexia nervosa? Behav Res Ther. 2012;50(3):180-90.

42. Horgan A, McCarthy G, Sweeney J. An evaluation of an online peer support forum for university students with depressive symptoms. Arch Psychiatr Nurs. 2013;27(2):84-9.

43. Goodyear-Smith F, Corter A, Suh H. Electronic screening for lifestyle issues and mental health in youth: a community-based participatory research approach. BMC Medical Informatics and Decision Making. 2016;16(1).

44. Whalen C, Moss D, Ilan AB, Vaupel M, Fielding P, Macdonald K, Cernich S, Symon J. Efficacy of TeachTown: Basics computer-assisted intervention for the Intensive Comprehensive Autism Program in Los Angeles Unified School District. Autism. 2010;14(3):179-97.

45. Rosa VO, Schmitz M, Moreira-Maia CR, Wagner F, Londero I, Bassotto CF, Moritz G, de Souza CDS, Rohde LAP. Computerized cognitive training in children and adolescents with attention deficit/hyperactivity disorder as add-on treatment to stimulants: feasibility study and protocol description. Trends Psychiatry Psychother. 2017;39(2):65-76.

46. Kollins SH, Bower J, Findling RL, Keefe R, Epstein J, Cutler AJ, White R, Aberle L, DeLoss D, Faraone SV. 2.40 A Multicenter, Randomized, Active-Control Registration Trial of Software Treatment for Actively Reducing Severity of ADHD (Stars-Adhd) to Assess the Efficacy and Safety of a Novel, Home-Based, Digital Treatment for Pediatric ADHD. Journal of the American Academy of Child & Adolescent Psychiatry. 2018;57(10):S172.

47. Waters AM, Zimmer-Gembeck MJ, Craske MG, Pine DS, Bradley BP, Mogg K. Look for good and never give up: A novel attention training treatment for childhood anxiety disorders. Behav Res Ther. 2015;73:111-23.

48. Lim CG, Lee TS, Guan C, Fung DS, Zhao Y, Teng SS, Zhang H, Krishnan KR. A brain-computer interface based attention training program for treating attention deficit hyperactivity disorder. PLoS One. 2012;7(10):e46692.

49. Crawford EA, Salloum A, Lewin AB, Andel R, Murphy TK, Storch EA. A Pilot Study of Computer-Assisted Cognitive Behavioral Therapy for Childhood Anxiety in Community Mental Health Centers. Journal of Cognitive Psychotherapy. 2013;27(3):221-34.

50. Holzer L, Urben S, Passini CM, Jaugey L, Herzog MH, Halfon O, Pihet S. A randomized controlled trial of the effectiveness of computer-assisted cognitive remediation (CACR) in adolescents with psychosis or at high risk of psychosis. Behav Cogn Psychother. 2014;42(4):421-34.

51. Olivet J, Haselden M, Piscitelli S, Kenney R, Shulman A, Medoff D, Dixon L. Results from a pilot study of a computer-based role-playing game for young people with psychosis. Early Interv Psychiatry. 2019;13(4):767-72.

52. Chapman R, Loades M, O'Reilly G, Coyle D, Patterson M, Salkovskis P. 'Pesky gNATs': Investigating the feasibility of a novel computerized CBT intervention for adolescents with anxiety and/or depression in a Tier 3 CAMHS setting. Cognitive Behaviour Therapist. 2016;9:<xocs:firstpage xmlns:xocs=""/>.

53. Klingberg T, Fernell E, Olesen PJ, Johnson M, Gustafsson P, Dahlström K, Gillberg CG, Forssberg H, Westerberg H. Computerized Training of Working Memory in Children With ADHD-A Randomized, Controlled Trial. Journal of the American Academy of Child & Adolescent Psychiatry. 2005;44(2):177--86.

54. Chukoskie L, Westerfield M, Townsend J. A novel approach to training attention and gaze in ASD: A feasibility and efficacy pilot study. Dev Neurobiol. 2018;78(5):546-54.

55. Silver M, Oakes P. Evaluation of a New Computer Intervention to Teach People with Autism or Asperger Syndrome to Recognize and Predict Emotions in Others. Autism. 2001;5(3):299-316.

56. Smith P, Scott R, Eshkevari E, Jatta F, Leigh E, Harris V, Robinson A, Abeles P, Proudfoot J, Verduyn C, Yule W. Computerised CBT for depressed adolescents: Randomised controlled trial. Behav Res Ther. 2015;73:104-10.

57. Brezinka V. Computer games supporting cognitive behaviour therapy in children. Clin Child Psychol Psychiatry. 2014;19(1):100-10.

58. Burns JM, Webb M, Durkin LA, Hickie IB. Reach Out Central: a serious game designed to engage young men to improve mental health and wellbeing. The Medical journal of Australia. 2010;192(11 Suppl):S27.

59. Romero NL. A Pilot Study Examining a Computer-Based Intervention to Improve Recognition and Understanding of Emotions in Young Children with Communication and Social Deficits. Res Dev Disabil. 2017;65:35-45.

60. Kuosmanen T, Fleming TM, Barry MM. The implementation of SPARX-R computerized mental health program in alternative education: Exploring the factors contributing to engagement and dropout. Children and Youth Services Review. 2018;84:176-84.

61. Dovis S, Van der Oord S, Wiers RW, Prins PJ. Improving executive functioning in children with ADHD: training multiple executive functions within the context of a computer game. a randomized double-blind placebo controlled trial. PLoS One. 2015;10(4):e0121651.

62. Mariano MA, Tang K, Kurtz M, Kates WR. Cognitive remediation for adolescents with 22q11 deletion syndrome (22q11DS): a preliminary study examining effectiveness, feasibility, and fidelity of a hybrid strategy, remote and computer-based intervention. Schizophr Res. 2015;166(1-3):283-9.

63. Bul KCM, Doove LL, Franken IHA, Oord SV, Kato PM, Maras A. A serious game for children with Attention Deficit Hyperactivity Disorder: Who benefits the most? PLoS One. 2018;13(3):e0193681.

64. Merry SN, Stasiak K, Shepherd M, Frampton C, Fleming T, Lucassen MF. The effectiveness of SPARX, a computerised self help intervention for adolescents seeking help for depression: randomised controlled non-inferiority trial. BMJ. 2012;344:e2598.

65. Kim MS, Blair KS, Lim KW. Using tablet assisted Social Stories to improve classroom behavior for adolescents with intellectual disabilities. Res Dev Disabil. 2014;35(9):2241-51.

66. Williams C, Wright B, Callaghan G, Coughlan B. Do Children with Autism Learn to Read more Readily by Computer Assisted Instruction or Traditional Book Methods?: A Pilot Study. Autism. 2002;6(1):71-91.

67. Kobak KA, Mundt JC, Kennard B. Integrating technology into cognitive behavior therapy for adolescent depression: a pilot study. Ann Gen Psychiatry. 2015;14:37.

68. Fleischmann RJ, Harrer M, Zarski AC, Baumeister H, Lehr D, Ebert DD. Patients' experiences in a guided Internet- and App-based stress intervention for college students: A qualitative study. Internet Interventions. 2018;12:130-40.

69. Jones DJ, Forehand R, Cuellar J, Parent J, Honeycutt A, Khavjou O, Gonzalez M, Anton M, Newey GA. Technology-enhanced program for child disruptive behavior disorders: development and pilot randomized control trial. J Clin Child Adolesc Psychol. 2014;43(1):88-101.

70. Kennard BD, Goldstein T, Foxwell AA, McMakin DL, Wolfe K, Biernesser C, Moorehead A, Douaihy A, Zullo L, Wentroble E, Owen V, Zelazny J, Iyengar S, Porta G, Brent D. As Safe as Possible (ASAP): A Brief App-Supported Inpatient Intervention to Prevent Postdischarge Suicidal Behavior in Hospitalized, Suicidal Adolescents. Am J Psychiatry. 2018;175(9):864-72.

71. Peters D, Davis S, Calvo RA, Sawyer SM, Smith L, Foster JM. Young People's Preferences for an Asthma Self-Management App Highlight Psychological Needs: A Participatory Study. J Med Internet Res. 2017;19(4):e113.

72. Edbrooke-Childs J, Edridge C, Averill P, Delane L, Hollis C, Craven MP, Martin K, Feltham A, Jeremy G, Deighton J, Wolpert M. A Feasibility Trial of Power Up: Smartphone App to Support Patient Activation and Shared Decision Making for Mental Health in Young People. JMIR Mhealth Uhealth. 2019;7(6):e11677.

73. Traber-Walker N, Metzler S, Gerstenberg M, Walitza S, Franscini M. SMARTPHONE APPLICATION "ROBIN": FEASIBILITY, ENGAGEMENT AND SATISFACTION OF A SMARTPHONE APPLICATION APPROACH TO SUPPORT TREATMENT OF (ATTENUATED) PSYCHOTIC SYMPTOMS IN ADOLESCENTS. Schizophrenia Bulletin. 2018;44(s1):S230-S.

74. Lattie EG, Ho J, Sargent E, Tomasino KN, Smith JD, Brown CH, Mohr DC. Teens Engaged in Collaborative Health: The Feasibility and Acceptability of an Online Skill-Building Intervention for Adolescents at Risk for Depression. Internet Interv. 2017;8:15-26.

75. Ho J, Corden ME, Caccamo L, Tomasino KN, Duffecy J, Begale M, Mohr DC. Design and evaluation of a peer network to support adherence to a web-based intervention for adolescents. Internet Interventions. 2016;6:50-6.

76. Whitehouse AJO, Granich J, Alvares G, Busacca M, Cooper MN, Dass A, Duong T, Harper R, Marshall W, Richdale A, Rodwell T, Trembath D, Vellanki P, Moore DW, Anderson A. A randomised controlled trial of an iPad-based application to complement early behavioural intervention in Autism Spectrum Disorder. J Child Psychol Psychiatry. 2017;58(9):1042-52.

77. Bekele ET, Lahiri U, Swanson AR, Crittendon JA, Warren ZE, Sarkar N. A step towards developing adaptive robot-mediated intervention architecture (ARIA) for children with autism. IEEE Trans Neural Syst Rehabil Eng. 2013;21(2):289-99.

78. Kumazaki H, Yoshikawa Y, Yoshimura Y, Ikeda T, Hasegawa C, Saito DN, Tomiyama S, An KM, Shimaya J, Ishiguro H, Matsumoto Y, Minabe Y, Kikuchi M. The impact of robotic intervention on joint attention in children with autism spectrum disorders. Mol Autism. 2018;9:46.

79. Schoenfelder E, Moreno M, Wilner M, Whitlock KB, Mendoza JA. Piloting a mobile health intervention to increase physical activity for adolescents with ADHD. Prev Med Rep. 2017;6:210-3.

80. Zhang L, Warren Z, Swanson A, Weitlauf A, Sarkar N. Understanding Performance and Verbal-Communication of Children with ASD in a Collaborative Virtual Environment. J Autism Dev Disord. 2018;48(8):2779-89.

81. Yuan SNV, Ip HHS. Using virtual reality to train emotional and social skills in children with autism spectrum disorder. London Journal of Primary Care. 2018;10(4):110-2.

82. Cai Y, Chia NK, Thalmann D, Kee NK, Zheng J, Thalmann NM. Design and development of a Virtual Dolphinarium for children with autism. IEEE Trans Neural Syst Rehabil Eng. 2013;21(2):208-17.

83. Gonzales R, Ang A, Murphy DA, Glik DC, Anglin MD. Substance use recovery outcomes among a cohort of youth participating in a mobile-based texting aftercare pilot program. Journal of Substance Abuse Treatment. 2014;47(1):20-6.
