# Supplementary material for: Engaging Children and Young People in Digital Mental Health Interventions: Systematic Review of Modes of Delivery, Facilitators, and Barriers
Source: J Med Internet Res. 2020 Jun 23;22(6):e16317. doi: 10.2196/16317 (PMC7381028; doi:10.2196/16317)
Supplement: Multimedia Appendix 3 [file jmir_v22i6e16317_app3.pdf]

#	Intervention description	Approach: features	Presenting symptoms/disorder	Purpose
1	Breathe is an internet-based CBT treatment program. The program includes online (CBT treatment modules) and Internet (e-mail support) based features as well as telephone support provided by a trained health care professional ('Anxiety Expert').	Website: videos, homework	Anxiety disorders	1*
2	Bounce Back Now (BBN) includes 4 modules to manage PTSD, depressions, ciagrette use and alcohol use; each module has screening questions for relevant symptoms (those without relevant symptoms could complete or skip a given module); modules were developed using evidence-based treatments for the respective conditions. Treatment approach is done through pychoeducation, exposure, coping, behavioural activiation, motivational-enhancement and CBT.	Website: text, graphics, anmisations, videos, and quizzes	Depression, PTSD, substance use	1, 4*
3	BRAVE for Children–ONLINE is adapted from a clinic-based, CBT anxiety treatment program (the BRAVE Program) and based on theoretical and empirical research relating to the psychosocial determinants of child anxiety.	Website: quizzes, pop-ups, homework tasks, automated reminders, profile set up, animations, sound clips, and interactive quizzes, weekly emails, games.	anxiety	1
4	Mood Mechanic Course, an internet-delivered cognitive behavioural therapy for young adults with anxiety and depression which consisted of four lessons delivered over 5 weeks.	Website: phone calls, emals, text messages	anxiety and depression	1
5	WebTIPS: tailored, innovative, web-based preoperative preparation program for children undergoing surgery and their parents. The children's website is tailored based upon children's trait anxiety and type of surgery and includes information provision, modeling, and coping skills through CBT.	Website: Games, video, audio, animations	anxiety	1

1- Provide trageted MH information to CYP based on health status or demographic; 2- Provide MH information to the general public; 3- Facilitate peer support network; 4- Facilitate personal health tracking

6	BRAVE for Teenagers-ONLINE: Standard CBT anxiety management strategies were used, including psychoeducation, relaxation training, recognition of the physiological symptoms of anxiety, cognitive strategies of coping self-talk and cognitive restructuring, graded exposure, problem solving, and self-reinforcement. The content, length, and number of session activities in the Internet program replicate those of the clinic-based session. Information is presented through interactive exercises and is followed by quizzes that check for correct understanding and provide personalized corrective or positive feedback through pop-up messages.	Website: Eye-catching graphics, sounds, games, and quizzes	meet criteria for a principal diagnosis of separation anxiety disorder, generalized anxiety disorder, social phobia, or specific phobia.	1
7	Internet delivered CBT for Social Anxiety Disorder.	Website: emails	SAD: Social Anxiety disorder	1
8	The Toolbox: a personalized app recommendation service available through the ReachOut.com website.	Website: email+text reminders	emotional, social and psychological well-being well-being	1
9	ICBT (internet-based cognitive behavioral therapy) was adapted for adolescents and involves 6-9 treatment modules.	Website: combined with additional telephone calls or face-to-face sessions if required.	Anxiety disorders	1
10	PRIME: Personalized real-time CBT intervention for motivational enhancement, a supportive online environment where persons select and document progress on small, self-determined goals in the domains of health/wellness, social relationships, creativity, and productivity.	Website: automated reminders, social networking	recent-onset schizophrenia spectrum disorders (SSDs).	1,3

1- Provide trageted MH information to CYP based on health status or demographic; 2- Provide MH information to the general public; 3- Facilitate peer support network; 4- Facilitate personal health tracking

11	CALM: Computer Assisted Learning for the Mind is a self-care website containing written information and audiofiles to promote good mental health and wellbeing. The website was divided into three sections: ‘managing stress, anxiety and depression’; ‘mental resilience’; and ‘finding meaning in life’ utilising CBT, mindfulness, positive psychology, gratitude, compassion.	Website: audiofiles	anxiety and depression	1
12	StudentBodies-Eating Disorders: designed to reduce eating disorder symptoms, related psychopathology, and weight and shape concerns. SB-ED consists of 10 weekly sessions and includes activities such as self-monitoring logs<br>and journal entries. It adopts CBT theory and techniques (behaviour modification, cognitive restructuring, increase awareness of thoughts/emotions, motivation and behaviour modifications, social support, problem solving, support and guidance).	Website, Text messages, discussion group	Subclinical eating disorders (AN, BN, binge eating disorder (BED), or purging disorder)	1,3*
13	SCOPE (Spectrum COmputerized PsychoEducation), internet-delivered, coach-guided, psychoeducational CBT intervention, is intended to serve as a first-line intervention for intellectually able older adolescents and young adults (16–25 years of age) diagnosed with ASD.	Website, Online/Internet : message function, video link options	autism spectrum disorder	1
14	Bite Back is an online positive psychology website for adolescents and uses a combination of interactive exercises and information across 9 positive psychology domains: gratitude, optimism, flow, meaning, hope, mindfulness, character strengths, healthy lifestyle, and positive relationships.	Website	Anxiety and depression	1,3

1- Provide trageted MH information to CYP based on health status or demographic; 2- Provide MH information to the general public; 3- Facilitate peer support network; 4- Facilitate personal health tracking

15	Breathe Eeasier Online (BEO): Online intervention devised to enhance social problem-solving skills and improve treatment adherence.	Website	Chronic respiratory illnesses	1,3
16	CA CIFFTA: Computer Assisted Culturally Informed and Flexible Family-Based Treatment for Adolescents (CIFFTA): 4-6 computer-based modules and 6-10 face-to-face sessions over 12 weeks based on motivational interviewing.	Website	Depression, conduct problems and family conflict	1
17	CATCH-IT (Competent Adulthood Transition with Cognitive-behavioral, Humanistic and Interpersonal Training) is a primary care/Internet-based "behavioral" vaccine intended for adolescents at elevated risk for depression. The intervention consists of 14 modules based on Behavioral Activation, CBT , Interpersonal Psychotherapy, and a community resiliency concept model.	Website	Depression	1
18	CCT (Compterized cognitive training): The training program employed a process-based approach to CCT,<br>in which cognitive improvement is driven by repetitive completion of the same type of task at varying degrees of difficulty and complexity.	Website	at least mild depressive symptoms defined as≥10<br>on the Hamilton Depression Rating Scale (HDRS).	1
19	CLIN-NET is a CBT service that provided half of the sessions delivered via the Internet. For the child program, 5 of the 10 sessions plus the 3-month booster session were adapted to Internet delivery,with the remaining sessions being conducted in the clinic.	Website	Anxiety	1
20	Grasp the Opportunity:Adaptation of CATCH-IT, an internet-based depression prevention intervention for early- and late-stage adolescents, utilized aspects of cognitive behavioral therapy (CBT), interpersonal therapy (IPT), behavioral activation (BA), and resiliency theory.	Website	Depression	1

1- Provide trageted MH information to CYP based on health status or demographic; 2- Provide MH information to the general public; 3- Facilitate peer support network; 4- Facilitate personal health tracking

21	MATE (Mindful Awareness Training and Evaluation): A 6-week online mindfulness training course.	Website	50% had previous mh disorder, mdd,anxiety,ptsd, bipolar	1,3
22	Meeky Mouse: a modified version of the original web-based CBT programme developed at the Hospital for Sick Children, Canada resulted in a web-based cognitive-behavioural therapy programme for the treatment of selective mutism. This involves a 14-week 'Meeky Mouse' programme consisting of eight training sessions, followed by six practice sessions (exposure using social skills training).	Website	selective mutism	1
23	MOMENTUM: designed to foster self-efficacy and increase positive emotions while providing mindfulness and social support through a purpose-built online social environment.	Website	Ultra High Risk (UHR) for psychosis criteria as assessed by the CAARMS	1,3
24	MoodGym: An interactive internet intervention consiting of 5 modules, based on CBT to prevent symptoms of depression and anxiety, contains exercises, quizzes and workbooks.	Website	Anxiety and depression	1
25	MYLO (Manage Your Life Online): a psychotherapy program that lends itself well to automation and application to computer-based self-help. MYLO could potentially provide an intervention that is effective for any problem a client is "stuck" with. It adopts CBT based _ Metod of Levels principles-Rogerian Psychotherapy	Website	DISTRESS: Depression, Anxiety and Stress	4
26	OCD? Not Me! is a fully automated eight-stage online CBT treatment program for young people (12–18 years) who are experiencing symptoms of OCD. The eight stages correspond to eight treatment modules, designed to be completed at a rate of one module per week.	Website	OCD	1

1- Provide trageted MH information to CYP based on health status or demographic; 2- Provide MH information to the general public; 3- Facilitate peer support network; 4- Facilitate personal health tracking

27	Online therapy programs	Website	not specified but the sample experienced mental health problems, including anxiety and worry, depression, anger, bullying and [illegible]	1
28	Reframe-IT comprised eight modules of CBT delivered over a 10-week intervention period. The eight modules cover each of the following topics: engagement and problem identification, emotional recognition and distress tolerance, identification of negative automatic thinking, behavioural activation—help seeking, behavioural activation—activity scheduling (including relaxation techniques), problem solving, and cognitive restructuring, and a wrap up session. This content was delivered via a series of video diaries.	Website	suicide-related behaviour (anxiety, depression, hopelessness)	1
29	SharpTalk: operated as both a support group and an online focus group.	Website	self-harm	3
30	Student Bodies: 8-week CBT internet-based program focused on reducing body weight and shape concerns.	Website	high-risk of eating disorder	1,4
31	StudentBodies2 - BED : a 16-week, Internet-facilitated, semi-structured program that incorporates cognitive-behavioral principles from the self-help manual for binge eating disorder, weight loss intervention, and hunger and satiety awareness skills.	Website	at risk for overweight, binge eating	1,4
30	VIA: CBT web-based relapse prevention program for anorexia nervosa.	Website	anorexia-nevrosa	1,4
31	www.losetheblues.ie : The Web site provided a forum to allow participants to offer peer support and and to disseminate information on depression and links to other sources of support.	Website	Depression	1,3

1- Provide trageted MH information to CYP based on health status or demographic; 2- Provide MH information to the general public; 3- Facilitate peer support network; 4- Facilitate personal health tracking

32	YouthCHAT, adaptations were made to eCHAT (electronic Case-finding and Help Assessment Tool) to develop a CBT youth programme for electronic screening and intervention for lifestyle risk factors and mental health issues.	Website	lifestyle issues<br>and mental health in youth	1,2*,4
33	TeachTown: a program that includes computer lessons and natural environment activities (Connection Activities) for developmental ages 2–7 years. The computer lessons offer a focused learning environment where the child works on the computer and completes lessons that incorporate the basic principles of Applied Behaviour Analysis.	Games: animations	Austism	1
34	ACTIVATE™: Active cognitive training – Computerized Cognitive Remediation Training (CCRT) (ACTIVATE™). CCRT comprises six different games that target neurocognitive functions, such as working memory, speed processing, sustained and divided attention, category formation and control inhibition. During the training, participants perform a wide range of cognitive tasks like memorizing sequences, completing patterns, task-switching and assigning objects into	Games	attention deficit/hyperactivity disorder (ADHD)	1,4
35	AKL-T01 is an investigational therapeutic cognitive training digital intervention for the treatment of attention and inhibitory control deficits in pediatric ADHD.	Games	ADHD	1

1- Provide trageted MH information to CYP based on health status or demographic; 2- Provide MH information to the general public; 3- Facilitate peer support network; 4- Facilitate personal health tracking

36	Attention Training Treatment (metacognitive therapy) involves 12 treatment sessions with each one including nine blocks of trials: four blocks of 20 trials, four blocks of 26 trials and one block of 40 trials (total 224 trials). Each trial consisted of either a 3x3 or a 4x4 picture array containing unpleasant background images (e.g. house on fire, person in hospital) and between one and three positive targets which are either ‘good’ targets (e.g., happy children; cute animals) or ‘calm’ targets (e.g., a vase; a book), which are less positive than ‘good’ targets but still positive to focus on given that not every situation will contain an explicitly pleasant stimulus to focus on.	Games	Anxiety disorders (separation anxiety disorder, generalized anxiety disorder, social phobia, specific phobia)	1
37	Brain-computer interface (BCI) includes training games using electroencephalogram (EEG). The BCI can quantify one’s attention level as measured by EEG waves, thereby allowing users to employ their attention to play these games directly.	Games	Attention deficit hyperactivity disorder	Training
38	Camp Cope-A-Lot (CCAL): interactive computer-assisted treatment program derived from the Coping Cat cognitive behavioral treatment approach for anxiety in youth. Throughout the program, participants followed along and participated with Charlie, a “camper” at CCAL, to learn coping strategies for anxiety and to overcome fears through exposures.	Games	anxiety symptoms	1
39	Captain's Log - Computer-assisted cognitive remediation (CACR) consists of five modules; the software consists of 35 multi-level “brain-training” exercises designed to develop and remediate attention, concentration, memory, eye–hand coordination, basic numeric concepts, problem-solving/reasoning skills, self-esteem and self-control.	Games	Psychosis or at High Risk of Psychosis	Development
40	OnTrack: computer-based role-playing game for young people with psychosis. The Game allows players to explore an imaginary world.	Games	FEP	1

1- Provide trageted MH information to CYP based on health status or demographic; 2- Provide MH information to the general public; 3- Facilitate peer support network; 4- Facilitate personal health tracking

41	Pesky gNATs' developed by O'Reilly & Coyle, 2015 is a seven level 3D computer game that provided a psychometric assessment of symptoms, supports a CBT intervention and coaches mindfulness and self-regulation skills for young people aged 9 -12 with anxiety and low mood.	Games	anxiety and /or depression	1
42	RoboMemo: a CBT based programme consisting of performing working memory tasks implemented in a computer program. The program included visuospatial tasks grid as well as verbal tasks.	Games	ADHD (diagnosis)	1
43	The cognitive training games are three colorful and engaging games with interesting sound effects. These games were designed with principles to train fixation, speed and accuracy of eye movements and control of visuo-spatial attention.	Games	diagnosis of ASD	Skills Training (fixation, speed and accuracy of eye movements and control of visuo-
44	The Emotion Trainer is a multimedia computer program. On each page of the program a digital photograph of a face, a scene or an object is shown, with a short text question and either two or four response buttons.	Game	autism or Asperger syndrome	1
45	Stressbusters, CBT approaches delivered via computer through the use of secure, interactive multimedia (animation and videos). In each session, the user: securely logs on; reports on homework and current mood; introduced to the topic of the session; chooses a video to watch of a depressed teenager (young actor) implementing the treatment technique; applies the technique to themselves through the use of interactivity; designs their own individualised homework based on that technique; and then logs out.	Computer: animation, video	Depression	1,4
46	Treasure Hunt is a game that corresponds to a certain step in cognitive-behavioural treatment, representing basic concepts of CBT for children. Thus, Treasure Hunt can be described as a broadband-CBT-game covering issues that are relevant in the treatment of various disorders.	Computer Games	wide range child mental health disorders	1

1- Provide trageted MH information to CYP based on health status or demographic; 2- Provide MH information to the general public; 3- Facilitate peer support network; 4- Facilitate personal health tracking

47	ROC, Reach Out Central is an internet-based “serious” game designed to improve the mental health and wellbeing of young people, particularly men. ROC is not a structured treatment; rather, the objectives of the game are to teach life skills,<br>such as communication, problem solving and optimistic thinking, in a virtual setting using real-life scenarios. Principles of behaviour change, social cognitive theory, the elaboration likelihood model and CBT are adopted.	Computer Game: text messages	mental health and wellbeing	1
48	The Transporters: consists of 15, five-minute animated episodes that each focus on an emotion or mental state. The 15 emotions<br>addressed in The Transporters are happy, sad, angry, afraid, disgusted, surprised, excited, tired, unfriendly, kind, sorry, proud, jealous, joking, and ashamed and targetted using emotion recognition training.	Computer Game: animation, quiz	communication<br>and social skills deficits (CSS)	1
49	SPARX-R is a CBT-based self-help intervention framed as a preventio**n** program targeting feelings of anger, stress and feeling low mood.	Computer Game (Offline)	targeting feelings of anger, stress and feeling low rather than depression	1, 2
50	Braingame Brian: a computerized, home-based executive functioning training, embedded in a game world and is named after its main character “Brian”. Brian is a young inventor who, throughout the game, helps and befriends the game-worlds inhabitants by creating increasingly elaborate inventions (e.g., a delivery-rocket for the grocery-store owner). Training tasks include: 1: working memory, 2 cognitive flexibility, 3 inhibition task.	Computer game	ADHD	1
51	COM: Challenging Our Minds involves cognitive tasks designed as games to improve skills across several domains including attention, executive function, memory, visual-spatial abilities, problem solving, and communication.<br>The COM system is completed on-line. Cognitive tasks are designed as	Computer Game	intellectual and behavioral deficits	4

1- Provide trageted MH information to CYP based on health status or demographic; 2- Provide MH information to the general public; 3- Facilitate peer support network; 4- Facilitate personal health tracking

52	Plan-It Commander: designed to improve functional outcomes in daily life with a primary focus on time management, planning/organizing and cooperation skills among children with ADHD through concepts of the self regulation model, social cognitive theory and learning theories.	Computer Game	ADHD	1
53	SPARX: an interactive fantasy game is designed to deliver cognitive behavioural therapy for the treatment of clinically significant depression.	Computer Game	Depressive symptoms	1,4
54	Social Stories (SS): computer-based ss picture symbols system combined with video modelling to teach social communicative skills.	Computer assisted: (Tablet): photos, stories	severe intellectual disabilities, problem behavior	1
55	CAI: Computer assissted instruction (CAI) includes book and card games scanned into a computer including sounds that children can read similar to hardcopy books.	Computer assisted	Autism	Facilitator

1- Provide trageted MH information to CYP based on health status or demographic; 2- Provide MH information to the general public; 3- Facilitate peer support network; 4- Facilitate personal health tracking

56	Technology enhanced CBT intervention for the treatment of adolescent depression consisting of (1) online therapist training<br>(2) in-session use of tablets for teaching clients CBT concepts and skills, and<br>(3) text messaging for between session<br>homework reminders and self-monitoring.	App; Text message reminders	Depression	1
57	StudyCare Stress is based on the principles of cognitive behavioral therapy and constitutes a modification of Get.On Stress, an online stress management intervention.	App: text, audia, video clips	stress-related symptoms	1
58	TE-HNC (Technology-Enhanced Helping the Noncompliant Child) includes the Helping the Noncompliant Child program, as well as smartphone components that were developed to enhance, rather than replace, clinic-based, therapist guided services based on the applied behaviour analysis model.	App: text reminders, video-calls, videos	Disruptive Behavior Disorders (DBDs)	1
59	ASAP: As Safe as Possible is a brief App-supported intervention consisting of four modules. The intervention includes a telephone app (BRITE) that promotes emotion regulation and provides access to a personalized safety plan during transition from inpatient care to outpatient care.	App: daily text messages, ability to upload vieos, web links, photos	recent suicidal ideation with a plan or intent or a recent suicide attempt; The majority of participants had a clinical diagnosis of major depression<br>(86.4%), often comorbid with an anxiety disorder (57.6%).	1
60	Kiss My Asthma: An Asthma Self--Management App for psychological support using self-determination theory.	App	Doctor-diagnosed asthma (self-reported)	1

1- Provide trageted MH information to CYP based on health status or demographic; 2- Provide MH information to the general public; 3- Facilitate peer support network; 4- Facilitate personal health tracking

61	Power Up is a transdiagnostic and transtherapeutic intervention to promote patient activation and shared decision-making among young people.	App : ability to upload vieos, web links, photos	Universal	2
62	Robin, a specific CBT smartphone application to support the therapy of adolescents with attenuated or full-blown psychotic symptoms. The smartphone application targets medication adherence, real-time symptom assessment and provides help coping with symptoms and stressful situations in daily life. The application is only used in combination with psychotherapy in our university hospital for child and adolescent psychiatry.	App	psychosis	1,4
63	TECH: Teen Enaged in Collaborative Health is a peer-networked internet delivered treatment intervention. CBT-based mood management tools, and peer networking features are embedded in TECH.	App	depression<br>and substance abuse	1,3,4
70	TECH: Teen Enaged in Collaborative Health is a peer-networked internet delivered treatment intervention. CBT-based mood management tools, and peer networking features are embedded in TECH.	App	depression (prevention)	1,3,4
64	TOBY (Therapy Outcomes By You) is an app-based learning curriculum designed for children and parents as a complement to early behavioural intervention based on applied behavior analysis.	App	ASD	1,4
65	ARIA: Adaptive Robot-Mediated Intervention Architecture involves the child sitting facing the human therapist or the robot administrator in their respective sessions and the human therapist and the robot presented the joint attention task using the hierarchical prompt protocol. A hierarchical prompt protocol was designed in a least-to most fashion with higher level of prompt provided on a need basis.	Robot	Autism Spectrum Disorder (ASD)	1

1- Provide trageted MH information to CYP based on health status or demographic; 2- Provide MH information to the general public; 3- Facilitate peer support network; 4- Facilitate personal health tracking

66	CommU is a 304 mm tall robot designed to prmote joint attention in children with ASD.	Robot	Autism - ASD	1
67	The Fitbit Flex & Facebook: collects data through its built-in accelerometer to provide proxy estimates of PA including steps, energy expended, and distance traveled. Users are asked to join a Facebook group to receive information and encouragement and digital badges are earned for completion of goals.	Digital Device: App, Website, email reminder	ADHD	3
68	CVE: Collaborative Virtual Environment where 2 geopgraphically distributed users can interact and communicate with each other in a shared environment. Several puzzle games promoting collaborative-communication and collaborative-action.	Virtual Reality: puzzle games	ASD and typically developed children	1
69	CAVE: Immersive VRE in a four-side Cave Automatic Virtual Environment (CAVE). Participants enter the CAVE individually and navigate the VRE with guidance and support. Six learning scenarious designed for relaxation, four training scenarios and one consolidation scenario.	Virtual Reality	ASD	1
70	VDI (virtual dolphin interaction): Virtual Reality - Dolphin-assisted CBT therapy for autism intervention and treatment.	Virtual Reality	autism spectrum disorder	1
71	ESQYIR (Educating & Supporting inquisitive Youth in Recovery) involves three types of text messaging: daily selfmonitoring texts, a daily wellness recovery tip, and substance abuse education and social support resource information on weekends.	Text Messages	substance abuse	1,4

1- Provide trageted MH information to CYP based on health status or demographic; 2- Provide MH information to the general public; 3- Facilitate peer support network; 4- Facilitate personal health tracking
