# Supplementary material for: Engaging Children and Young People in Digital Mental Health Interventions: Systematic Review of Modes of Delivery, Facilitators, and Barriers
Source: J Med Internet Res. 2020 Jun 23;22(6):e16317. doi: 10.2196/16317 (PMC7381028; doi:10.2196/16317)
Supplement: Multimedia Appendix 4 [file jmir_v22i6e16317_app4.pdf]

	First author, year	Intervention description	Study's Aim	Country	Study type & Method	Participants' age (years)	Sample size	Retention	Recruitment strategy used	MMAT Score
1	Wozney, 2015	Breathe is an internet-based CBT treatment program. The program includes online (CBT treatment modules) and Internet (e-mail support) based features as well as telephone support provided by a trained health care professional	To explore the usability of Internet-based component of the Breathe program in terms of learnability, efficiency, the frequency and	Canada	Mixed-methods	15 - 24 years	9	88.90	Clinicians and young people were recruited through a national contact (e-mail) list maintained by the National Infant, Child and Youth Mental Health Consortium in Canada	2
2	Price, 2015	Bounce Back Now (BBN) includes 4 modules to manage PTSD, depressions, ciagrette use and alcohol use; each module has screening questions for relevant symptoms (those without relevant symptoms could complete or skip a given module); modules were developed using evidence-based treatments for the respective conditions. Treatment approach is	To determine the proportion of adolescents, located in nonurban and urban areas, affected by tornados that accessed and completed a web-based treatment	USA	RCT	M=14.5, SD=1.7	2000	79.35	Specific regions were targetted using the coordinates of areas exposed to a series of tornados that occurred 4 months prior to recruitment; cenesus block IDs were assigned to the radiius around each coordainte to serve as the sampling strata. Households were recruited by phone or email.	2
3	March, 2009	BRAVE for Children–ONLINE is adapted from a clinic-based, CBT anxiety treatment program (the BRAVE Program) and based on theoretical and empirical research relating to the psychosocial determinants of child anxiety.	To evaluate the efficacy of an Internet-based cognitive-behavioral therapy approach to the treatment of child anxiety disorders	Australia	RCT	M=9.45, SD = 1.37	73	93.65	Children were referred to the program by parents, teachers, guidance officers, and other mental health professionals in response to media releases and information packages sent to schools.	4
4	Johnston, 2014	Mood Mechanic Course, an internet-delivered cognitive behavioural therapy for young adults with anxiety and depression which consisted of four lessons delivered over 5 weeks.	To examine the efficacy and acceptability of a new therapist-guided Internet-delivered treatment for young adults with symptoms	New Zealand	Quantitative	18–24 years	56	77.78	Interested people applied to participate in the study through a clinical research website.	3
5	Fortier, 2015	WebTIPS: tailored, innovative, web-based preoperative preparation program for children undergoing surgery and their parents. The children's website is tailored based upon children's trait anxiety and type of surgery and includes information provision, modeling	To conduct formative evaluation and test the preliminary efficacy of a newly developed web-based, tailored behavioral preparation program	USA	Mixed-methods	2-7 years	82	100.00	Researchers targetted parent-child dyads scheduled to undergo outpatient surgery and general anesthesia at Children's Hospital of Orange County or Yale-New Haven Children's Hospital between August 2011 and August 2012.	3

6	Anderson, 2012	BRAVE for Teenagers-ONLINE: Standard CBT anxiety management strategies were used, including psychoeducation, relaxation training, recognition of the physiological symptoms of anxiety, cognitive strategies of coping self-talk and cognitive restructuring, graded exposure, problem solving, and self-reinforcement. The content, length, and number of session activities in the Internet program replicate those of the clinic-based session. Information is presented through interactive	To compare the relative strength of working alliance in online versus clinic delivery of CBT for anxiety in youth	Australia	RCT	12 to 18 years (M 13.91, SD 1.56)	73	NK	Referrals from guidance officers, mental health professionals, and self-referrals following media publicity.	2
7	Tillfors, 2011	Internet delivered CBT for Social Anxiety Disorder.	To evaluate the effectiveness of an internet-delivered CBT program for	Sweden	RCT	M=16.5,SD=1.6	19	94.74	Regional newspaper artciles, school staff, advertisements in the high schools.	2
8	Bidargaddi, 2017	The Toolbox: a personalized app recommendation service available through the ReachOut.com website.	To assess the efficacy of a Web-based self-guided app recommendation service ("The Toolbox") in improving the well-being of young Australians	Australia	RCT	16-25 years (M=23)	387	49.87	Web-based paid advertisements were placed on Facebook, Twitter, YouTube, and Google AdWords. In addition to paid advertisements, links to the study site were provided to 39 organizations and educational institutions frequently visited by young people from different backgrounds to promote via their social media channels (Facebook, Twitter). Community-based organizations such as schools, universities, sporting clubs, and	3
9	Silfvernagel, 2015	ICBT (internet-based cognitive behavioral therapy) was adapted for adolescents and involves 6-9 treatment modules.	To examine the effects of tailored internet-based CBT for adolescents	Sweden	Quantitative	M=16.8	11	72.73	Referal routes at clinic (self-referral, parent, doctor) OR through website, brochures and information sessions.	3

10	Schlosser, 2018	PRIME: Personalized real-time CBT intervention for motivational enhancement, a supportive online environment where persons select and document progress on small, self-determined goals in the domains of health/wellness, social relationships, creativity, and productivity.	To compare the efficacy of PRIME intervention to a treatment-as-usual/waitlist control group	USA	RCT	16 - 36 years: PRIME group: M=24.32 (SD=2.6) Control group:	43	93.02	Participants were recruited remotely using Craigslist, online message boards, and flyers posted in clinics and doctor's offices. Study investigators also listed the study on the UCSF School of Medicine Clinical Trials website and the lab website, and directly contacted other research labs. Interested participants	4
11	Moir, 2015	CALM: Computer Assisted Learning for the Mind is a self-care website containing written information and audiofiles to promote good mental health and wellbeing. The website was divided into three sections: 'managing stress, anxiety and depression'; 'mental resilience'; and 'finding meaning in life' utilising CBT, mindfulness, positive [illegible]	To develop an evidence-based self-help website designed to improve mental health amongst medical students. To assess the proportion, demographics and mental health of students who chose to use	New Zealand	Quantitative	M=21.1; SD=3.2	321	24.92	Weblink was sent out to the an entire cohort/class of students.	2
12	Saekowa, 2015	StudentBodies-Eating Disorders: designed to reduce eating disorder symptoms, related psychopathology, and weight and shape concerns. SB-ED consists of 10 weekly sessions and includes activities such as self-monitoring logs and journal entries. It adopts CBT theory and techniques (behaviour modification, cognitive restructuring, increase awareness of thoughts/emotions, motivation and behaviour modifications, social	To examine the feasibility, acceptability, and short-term efficacy of a 10-week online intervention	USA	RCT	18–25 years	65	68.33	Recruited through print and online advertisements, university list-serve emails, and classroom and dorm presentations. Residential education staff and student health services staff also directed students to the study. Additionally, students learned about the study through the Healthy Body Image Program, a comprehensive online eating disorders screening and prevention	4
13	Backman, 2018	SCOPE (Spectrum COmputerized PsychoEducation), internet-delivered, coach-guided, psychoeducational CBT intervention, is intended to serve as a first-line intervention for intellectually able older adolescents and young adults (16–25 years of age) diagnosed with ASD	To evaluate the new internet-delivered, psychoeducational intervention for adolescents and young adults with ASD	Sweden	Quantitative	16–25 years	29	82.14	Information about the study was disseminated at various Habilitation & Health centers, at psychiatric outpatient units, as well as at private health care providers, and interested individuals were directed to the study's websites to sign up.	3

14	Manicavasagar, 2014	Bite Back is an online positive psychology website for adolescents and uses a combination of interactive exercises and information across 9 positive psychology domains: gratitude, optimism, flow, meaning, hope, mindfulness, character strengths, healthy lifestyle, and positive relationships.	To examine the feasibility of the online delivery of a youth positive psychology program to improve the well-being and mental health outcomes of Australian youth	Australia	RCT	M=15	235	65.50	Promotional information packs advertising the How Do You View the World Study were disseminated via mail and email and included a letter to the organization, principal, and/or school counselor that detailed the rationale, requirements, and participation incentives of the study, and a series of flyers promoting the study to young people. Organizations were asked to distribute the flyers or directly promote	3
15	Petsky, 2010	Breathe Eeasier Online (BEO): Online intervention devised to enhance social problem-solving skills and improve treatment adherence.	To test the intervention to improve the sense of well-being, social problem-solving skills, treatment compliance	Australia	RCT	10–17 years	42	47.62	NK	3
16	Santisteban, 2017	CA CIFFTA: Computer Assisted Culturally Informed and Flexible Family-Based Treatment for Adolescents (CIFFTA): 4-6 computer-based modules and 6-10 face-to-face sessions over 12	To test the efficacy of a computer-assisted treatment for behaviour problems and family conflict	USA	RCT	M=13.6, SD=1.17	80	67.50	Referrals through school counsellors and community agencies.	3
17	Landback, 2009	CATCH-IT (Competent Adulthood Transition with Cognitive-behavioral, Humanistic and Interpersonal Training) is a primary care/Internet-based “behavioral” vaccine intended for adolescents at elevated risk for depression. The intervention consists of 14 modules based on Behavioral Activation, CBT , Interpersonal	To revisit and improve the initial version of the CATCH-IT Internet-based depression prevention intervention. To change the intervention from a content-rich Internet self-help book to an aesthetically engaging	USA	Quantitative	14-21 years (M= 18; SD=2)	25	NK	Patients reporting depressed mood, anhedonia, irritability during their routine check-up or illness related visit at their primary care clinic were informed of the study.	4

18	Saulsberry, 2013	CATCH-IT (Competent Adulthood Transition with Cognitive-behavioral, Humanistic and Interpersonal Training) is a primary care/Internet-based “behavioral” vaccine intended for adolescents at elevated risk for depression. The intervention consists of 14 modules based on Behavioral [illegible]	To compare two forms of primary care provider (PCP) engagement (motivational interview [MI] and brief advice [BA]) for adolescents using the primary care/Internet-based	USA	RCT	M= 17.26;SD=1.85	84	70.00	Recruited primary care sites by approaching five major healthcare organizations and then approaching physicians within those organizations.	3
19	Gladstone, 2014	CATCH-IT (Competent Adulthood Transition with Cognitive-behavioral, Humanistic and Interpersonal Training) is a primary care/Internet-based “behavioral” vaccine intended for adolescents at elevated risk for depression. The intervention consists of 14 modules based on Behavioral Activation, CBT , Interpersonal Psychotherapy, and a community	To examine intervention variables that predict favorable changes in depressive symptoms at 6- to 8-week follow-up in at-risk adolescents who participated in a primary care, Internet-based	USA	Quantitative	14 - 21 years (M=17.5, SD=2.04)	83	83.13	Adolescents with primary care visits at participating clinics were screened for risk of disorder (subthreshold depressive symptoms) and given information for participation in the study.	2
20	Iloabachie, 2011	CATCH-IT (Competent Adulthood Transition with Cognitive-behavioral, Humanistic and Interpersonal Training) is a primary care/Internet-based “behavioral” vaccine intended for adolescents at elevated risk for depression. The intervention consists of 14 modules based on Behavioral Activation, CBT , Interpersonal Psychotherapy, and a community	To understand the experience of adolescents involved in the Internet-based intervention for depression (CATCH-IT), as well as the experiences of their parents while they were	USA	Mixed-methods	14 - 21 years	83	85.71	Primary care sites by approaching five major health care organizations and then approached physicians within those organizations.	4
21	Van Voorhees, 2009	CATCH-IT (Competent Adulthood Transition with Cognitive-behavioral, Humanistic and Interpersonal Training) is a primary care/Internet-based “behavioral” vaccine intended for adolescents at elevated risk for depression. The intervention consists of 14 modules based on Behavioral Activation, CBT , Interpersonal	To determine how two different forms of primary care physician engagement, brief advice (BA) versus motivational interview (MI), could enhance participation outcomes in an Internet- [illegible]	USA	RCT	14–21 years	83	92.68	Primary care sites by approaching five major health care organizations and then approached physicians within those organizations.	3

22	Van Voorhees, 2009	CATCH-IT (Competent Adulthood Transition with Cognitive-behavioral, Humanistic and Interpersonal Training) is a primary care/Internet-based "behavioral" vaccine intended for adolescents at elevated risk for depression. The intervention consists of 14 modules based on Behavioral Activation, CBT , Interpersonal Psychotherapy, and a community resiliency concept model.	To evaluate two approaches with varying time and complexity in engaging adolescents with an Internet-based preventive intervention for depression in primary care	USA	RCT	14–21 years	83	96.42	Primary care sites by approaching five major health care organizations and then approached physicians within those organizations.	5
23	Motter, 2019	CCT (Compterized cognitive training): The training program employed a process-based approach to CCT, in which cognitive improvement is driven by repetitive completion of the same type of task at varying degrees of difficulty and	To test the proposed executive functioning/processing speed mechanism of action of Computerized Cognitive Training on depressive	USA	RCT	18–29 years (M = 21.0, SD=3.7)	46	76.00	Participants were recruited via online advertisements, flyers in the community, and electronic mailing lists of various college/university counseling centers, academic departments, and social groups.	2
24	Spence, 2006	CLIN-NET is a CBT service that provided half of the sessions delivered via the Internet. For the child program, 5 of the 10 sessions plus the 3-month booster session were adapted to Internet delivery,with the remaining sessions being conducted in the	To explore the feasibility and efficacy of partially delivering CBT for the child anxiety disorders via the Internet	Australia	RCT	7 - 14 years	72	90.27	Referred from mental health clinics and school counselors.	**4**
25	Sobowale, 2013	Grasp the Opportunity:Adaptation of CATCH-IT, an internet-based depression prevention intervention for early- and late-stage adolescents, utilized aspects of cognitive behavioral therapy (CBT), interpersonal therapy (IPT), behavioral activation (BA), and resiliency theory.	To develop a revised intervention of the US version of the CATCH-IT program for Chinese adolescents in order to improve resiliency in youth at risk for depression	Hong Kong	Qualitative	15-24 years	16	NK	Participants were recruited by e-mail and personal contact from the University of Hong Kong and the Wah Yan College and a secondary school on Hong Kong Island.	**5**
26	Monshat, 2012	MATE (Mindful Awareness Training and Evaluation): A 6-week online mindfulness training course.	To involve young people in designing an online Mindfulness Training	Australia	Qualitative	16-26 years (M=22)	20	NK	Online advertisements	5

27	Ooi, 2012	Meeky Mouse: a modified version of the original web-based CBT programme developed at the Hospital for Sick Children, Canada resulted in a web-based cognitive-behavioural therapy programme for the treatment of selective mutism. This involves a 14-week 'Meeky Mouse' programme consisting of eight training [illegible]	To examine the use of a web-based CBT programme among Singaporean children diagnosed with selective mutism	Singapore	Mixed-methods	6-11 years (M=9; SD=2.35)	5	100.00	Referred to the study by their attending child psychiatrist.	3
28	Alvarez-Jimenez, 2018	MOMENTUM: designed to foster self-efficacy and increase positive emotions while providing mindfulness and social support through a purpose-built online social environment.	To describe the theoretical basis and therapeutic targets of a novel intervention targeting social functioning in UHR young people and to [illegible]	Australia	Mixed-methods	M = 20.3; SD = 3.4	14	92.86	Eligible participants were oriented to the system by the study coordinator a research clinic.	4
29	Neil, 2009	MoodGym: An interactive internet intervention consiting of 5 modules, based on CBT to prevent symptoms of depression and anxiety, contains exercises, quizzes [illegible]	To investigate adherence	Australia	Quantitative	3 - 17 years	8207	15.79	Participants at school were asked to log in while commuty sample self-referred by logging into the moodgym website	5
30	Lillevoll, 2014	MoodGym: An interactive internet intervention consiting of 5 modules, based on CBT to prevent symptoms of depression and anxiety, contains exercises, quizzes [illegible]	To evaluate the feasibility and efficacy of disseminating a self-directed internet-based mental	Norway	RCT	Control group: M=16.78 MoodGYM	707	71.15	Participants were recruited through visits of the research group to the four participating schools.	2
31	O'Kearney, 2009	MoodGym: An interactive internet intervention consiting of 5 modules, based on CBT to prevent symptoms of depression and [illegible]	To test effectiveness of a cognitive behaviour therapy Internet program for [illegible]	Australia	Quantitative	15-16 years	157	71.34	NK	2
32	O'Kearney, 2006	MoodGym: An interactive internet intervention consiting of 5 modules, based on CBT to prevent symptoms of depression and anxiety, contains exercises, quizzes	To test effectiveness of a cognitive behaviour therapy Internet program for [illegible]	Australia	Quantitative	15-16 years	78	79.66	Students were informed verbally about the study and provided with written information.	4
33	Calear, 2013	MoodGym: An interactive internet intervention consiting of 5 modules, based on CBT to prevent symptoms of depression and anxiety, contains exercises, quizzes [illegible]	To investigate adolescent adherence to an Internet-based depression prevention program in schools to	Australia	RCT	12-17 years (M=14.34; SD=0.75)	1477	87.80	NK	3

34	Gaffney, 2014	MYLO (Manage Your Life Online): a psychotherapy program that lends itself well to automation and application to computer-based self-help. MYLO could potentially provide an intervention that is effective for any problem a client is “stuck” with. It adopts CBT based	To test the efficacy of MYLO	UK	RCT	18–32 years (M = 21.4; SD = 3.1)	48	87.50	Advertisement on the university SONA system and volunteering website, recruitment posters and word of mouth.	4
35	Anderson, 2017	OCD? Not Me! is a fully automated eight-stage online CBT treatment program for young people (12–18 years) who are experiencing symptoms of OCD. The eight stages correspond to eight treatment modules, designed to be	To describe the key lessons learned in the process of developing and evaluating the first fully-automated iCBT program for young	Australia	NA	12-18 years	NK	NK	NK	NA
36	Sweeney, 2019	Online therapy programs	To explore adolescents' perceptions of online therapies, including	Australia	Quantitative	13-18 years (M=16.98; SD =1.32)	217	NK	Facebook posts on youth interest groups and advertising through secondary schools	3
37	Hetrick, 2017	Reframe-IT comprised eight modules of CBT delivered over a 10-week intervention period. The eight modules cover each of the following topics: engagement and problem identification, emotional recognition and distress tolerance, identification of negative automatic thinking, behavioural activation—help seeking, behavioural activation—activity scheduling (including relaxation techniques), problem solving, and cognitive restructuring, and a wrap	To test the effectiveness of an internet-based CBT program (Reframe-IT) in reducing suicide-related behaviours, depression, anxiety, hopelessness and improving problem solving and cognitive and behavioural skills in school students with suicide-related behaviours	Australia	RCT	13 -19 years	50	78.00	All secondary schools in the study catchment area were invited to participate.	5
38	Jones, 2011	SharpTalk: operated as both a support group and an online focus group.	To explore what young people who self-harm think about online self-harm discussion forums	UK	Qualitative	16-25 years (M = 19)	95	59.74	Announcements on existing online forums & Professionals and final-year students in health and social care disciplines were recruited by emails and advertisements in two universities.	2

39	Kass, 2013	Student Bodies: 8-week CBT internet-based program focused on reducing body weight and shape concerns.	To investigate whether a guided discussion group improved program efficacy in reducing weight and shape concerns associated with participation in the 8-week Student Bodies	USA	RCT	18-26 years	151	73.14	Flyers, referrals from campus health centers, e-mail advertisements, word to mouth.	4
40	Jones, 2008	StudentBodies2 - BED : a 16-week, Internet-facilitated, semi-structured program that incorporates cognitive-behavioral principles from the self-help manual for binge eating disorder, weight loss intervention, and hunger and satiety awareness skills.	To investigate the use of an Internet-facilitated intervention for weight maintenance and binge eating in adolescents	USA	RCT	M= 15.1; SD= 1.0	105	88.57	Flyers and presentations in health education and physical education classes at cooperating high schools. Additional recruitment presentations were conducted at student rallies, freshmen orientation, and parent back-to-school night.	3
41	Fischter, 2012	VIA: CBT web-based relapse prevention program for anorexia nervosa.	To assess an internet-based programe for relapse prevention for anorexia-nevrosa	Germany	RCT	M=23.8; SD=6.6 M=24.1; SD=5.6	258	75.78	Recruited through one of 8 hospitals in Germany providing inpatient services and psychotherapy for eating disorders	4
42	Horgan, 2013	www.losetheblues.ie : The Web site provided a forum to allow participants to offer peer support and and to disseminate information on depression and links to other sources of support.	To develop and pilot test an online peer support intervention for students experiencing depressive symptoms	Ireland	Mixed-methods	18-24 years (M = 20.6; SD= 1.8)	118	NK	Participants were recruited from a University in Ireland. An e-mail advertising the study was sent to all students on three occasions.	4
43	Goodyear-Smith, 2016	YouthCHAT, adaptations were made to eCHAT (electronic Case-finding and Help Assessment Tool) to develop a CBT youth programme for electronic [illegible]	To tailor the YouthCHAT package for use in a clinic catering for disadvantaged youth, assess its	New Zealand	Quantitative	< 25 years	30	NK	YouthCHAT was implemented in a health clinic colocated at a low-decile school with a high Māori population	5

44	Whallen, 2010	TeachTown: a program that includes computer lessons and natural environment activities (Connection Activities) for developmental ages 2–7 years. The computer lessons offer a focused learning environment where the child works on the computer and completes lessons that incorporate the basic principles of Applied	To assess the usefulness of the program in a school setting, where CAI programs for students with special needs are in short supply	USA	RCT	3 - 6 years	47	68.18	NK	3
45	de Oliveira Rosa, 2017	ACTIVATE™: Active cognitive training – Computerized Cognitive Remediation Training (CCRT) (ACTIVATE™). CCRT comprises six different games that target neurocognitive functions, such as working memory, speed processing, sustained and divided attention, category formation and control inhibition. During the training, participants perform a wide range of cognitive tasks like [illegible]	To explore the feasibility of implementing a computerized cognitive training program for ADHD, describe its main characteristics and potential efficacy in a small pilot study	Brazil	RCT	10-12-years (M= 10.83; SD= 0.75)	6	83.33	Patients invited to participate in the trial during their regular attendance at ProDAH. At this moment, the study protocol was explained to parents and participants to assess their interest and the feasibility of maintaining regular face-to-face training sessions. When the invitation was accepted, parents and [illegible]	2
46	Kollins, 2018	AKL-T01 is an investigational therapeutic cognitive training digital intervention for the treatment of attention and inhibitory control deficits in	To present primary efficacy and safety results of the randomized Software Treatment for Actively	USA	RCT	8-12 years (M= 9.7; SD = 1.3)	348	85.60	NK	2
47	Waters, 2015	Attention Training Treatment (metacognitive therapy) involves 12 treatment sessions with each one including nine blocks of trials: four blocks of 20 trials, four blocks of 26 trials and one block of 40 trials (total 224 trials). Each trial consisted of either a 3x3 or a 4x4 picture array containing unpleasant background images (e.g. house on fire, person in hospital) and between one and three positive targets which are either 'good' targets (e.g., happy children; cute animals) or 'calm'	To test of efficacy for a novel form of attention training towards positive stimuli for anxious children using visual-search ABMT supplemented with distinctive verbalization techniques designed to increase children's learning, memory consolidation, and engagement.	Australia	RCT	Intervention: M=9<br>Control: M=8.5	59	88.14	Parents responded to study advertisements circulated through local schools, paediatricians, GPs, other mental health and community health agencies, and local newspapers.	4

48	Lim, 2010	Brain-computer interface (BCI) includes training games using electroencephalogram (EEG). The BCI can quantify one's attention level as measured by EEG waves, thereby allowing users to employ their attention to play these games directly.	To investigate the efficacy and feasibility of using our BCI-based training programme to treat inattentive symptoms of ADHD	Singapore	Quantitative	7-12 years	20	80.00	Participants who were seen at the clinic and diagnosed to have either the combined or inattentive subtype of ADHD based on the Diagnostic and Statistical Manual of Mental Disorders, Fourth Edition (DSM-IV) were recruited.	4
49	Crawford, 2013	Camp Cope-A-Lot (CCAL): interactive computer-assisted treatment program derived from the Coping Cat cognitive behavioral treatment approach for anxiety in youth. Throughout the program, participants followed along and participated with Charlie, a "camper" at CCAL, to	To investigate effectiveness, feasibility, and acceptability of a Computerized CBT program in three Community Mental Health Centres	USA	Quantitative	7- 13 years (M=10.1; SD=1.9)	17	88.24	Families were referred within each CMHC for study participation, as well as through presentations to community providers and study flyers/brochures.	4
50	Holzer, 2014	Captain's Log - Computer-assisted cognitive remediation (CACR) consists of five modules; the software consists of 35 multi-level "brain-training" exercises designed to develop and remediate attention, concentration, memory, eye–hand coordination, basic numeric concepts, problem-	To investigate the effectiveness of a computer-assisted cognitive remediation (CACR) program in adolescents with psychosis or at high risk	Switzerland	RCT	M= 15.4	32	87.5%	Participants were recruited from the Day Care Unit for Adolescents (DCUA) while they were outpatients.	5
51	Olivet, 2018	OnTrack: computer-based role-playing game for young people with psychosis. The Game allows players to explore an imaginary world.	To develop a prototype computer-based role-playing game (RPG) designed for young people who have experienced first episode of	USA	Mixed-methods	M= 19.6;SD = 2.4	20	NK	Clinicians at a clinic identified potential participants and provided information about the study.	5
52	Chapman, 2016	Pesky gNATs' developed by O'Reilly & Coyle, 2015 is a seven level 3D computer game that provided a psychometric assessment of symptoms, supports a CBT intervention and coaches mindfulness and self-regulation	To pilot the feasibility and acceptability of using a novel cCBT intervention	UK	Mixed-methods	13-16 years	11	100.00	Families on a waiting list for CBT were contacted by site's local collaborators.	4

53	Klingberg, 2004	RoboMemo: a CBT based programme consisting of performing working memory tasks implemented in a computer program. The program included visuospatial tasks grid as well as	To investigate the effect of improving Working Memory (WM) by computerized, systematic practice of WM	Sweden	RCT	7 - 12 years	53	83.02	Referral sources included pediatricians, child psychiatrists, and special teachers in schools.	**3**
54	Chukoskie, 2017	The cognitive training games are three colorful and engaging games with interesting sound effects. These games were designed with principles to train fixation, speed and accuracy of eye movements	To demonstrate the feasibility and preliminary efficacy of using gaze-contingent video games for low cost in home training of	USA	Quantitative	11-16 years (M= 13.9)	8	75.00	NK	3
55	Silver, 2001	The Emotion Trainer is a multimedia computer program. On each page of the program a digital photograph of a face, a scene or an object is shown, with a short text question and either two or four response buttons	To evaluate a new computer program designed to teach people with autistic spectrum disorders to better recognize and predict	UK	RCT	M = 14	24	90.91	NK	3
56	Smith, 2015	Stressbusters, CBT approaches delivered via computer through the use of secure, interactive multimedia (animation and videos). In each session, the user: securely logs on; reports on homework and current mood; introduced to the topic of the session; chooses a video to watch of a depressed teenager (young actor) implementing the treatment technique; applies the technique	To test the efficacy of a Computerised-CBT programme for depression in young people	UK	RCT	12-16 years	112	98.21	Young persons at a secondary school were invited to participate.	4
57	Brezinka, 2014	Treasure Hunt is a game that corresponds to a certain step in cognitive-behavioural treatment, representing basic concepts of CBT for children. Thus, Treasure Hunt can be described as a broadband-CBT-game covering issues that are relevant in the treatment of various disorders.	To evaluate the applicability and appropriateness of the game	Switzerland, Germany, Belgium, the Netherland, Egypt	Quantitative	M = 10.35; SD 1.86	339	NK	Recruitment via email invitations.	4

58	Burns, 2010	ROC, Reach Out Central is an internet-based “serious” game designed to improve the mental health and wellbeing of young people, particularly men. ROC is not a structured treatment; rather, the objectives of the game are to teach life skills, such as communication, problem solving and optimistic thinking, in a virtual setting using real-life scenarios. Principles of behaviour	To develop Reach Out Central, a serious game that draws on the principles of cognitive behaviour theory	Australia	Quantitative	18–25 years	266	NK	Advertorials were posted on six gaming, entertainment, social and community youth websites. Specific male youth-oriented magazines were targeted, including Zoo Weekly, Alpha, Waves, Rolling Stone, XBox and FHM. The campaign was advertised in print and the popular press, including 1500 bus and tram interior advertisements nationally	2
59	Romero, 2017	The Transporters: consists of 15, five-minute animated episodes that each focus on an emotion or mental state. The 15 emotions addressed in The Transporters are happy, sad, angry, afraid, disgusted, surprised, excited, tired,	To investigate whether computer based instruction helps students improve ER ability in computer based	USA	Quantitative	4 -8 years	4	75.00	The primary researcher recruited participants from three communication and social skills deficits (CSS) classrooms at a selected site.	2
60	Kuosmanen, 2018	SPARX-R is a CBT-based self-help intervention framed as a prevention program targeting feelings of anger, stress and feeling	To examine factors affecting the implementation of SPARX-R cCBT in the context	Ireland	Mixed-methods	15–20 years (M= 17.32)	28	93.33	In each Youthreach Center, the program coordinator selected one class of approximately eight students to	3
61	Dovis, 2015	Braingame Brian: a computerized, home-based executive functioning training, embedded in a game world and is named after its main character “Brian”. Brian is a young inventor who, throughout the game, helps and befriends the game-worlds inhabitants by creating increasingly elaborate inventions (e.g., a delivery-rocket for the grocery-store owner).	To test whether improving executive functioning in children with ADHD within the context of a computer game has an effect on symptoms	Netherlands	RCT	8-12 years	89	93.26	Recruited from 14 outpatient mental-healthcare centers.	5

62	Mariano, 2015	COM: Challenging Our Minds involves cognitive tasks designed as games to improve skills across several domains including attention, executive function, memory, visual-spatial abilities, problem solving, and communication. The COM system is completed on-line. Cognitive tasks are designed as games involving a standardized sequence of computerized cognitive exercises intended to improve skills across several	To investigate the efficacy, feasibility, and fidelity of a remote, hybrid strategy, computerized CR program in youth	USA	Quantitative	12 - 15 years	22	100.00	Recruited through parent support groups and referrals from clinicians.	4
63	Bul, 2018	Plan-It Commander: designed to improve functional outcomes in daily life with a primary focus on time management, planning/organizing and cooperation skills among children	To identify which subgroups of children with Attention Deficit Hyperactivity Disorder (ADHD) benefitted [illegible]	Europe (Belgium, The Netherla [illegible]	RCT	M=9.90; SD = 1.26	143	89.40	NK	3
64	Merry, 2012	SPARX: an interactive fantasy game is designed to deliver cognitive behavioural therapy for the treatment of clinically significant depression.	To evaluate whether a new computerised cognitive behavioural therapy intervention (SPARX, Smart,	New Zealand	RCT	12-19 years	187	86.00	Young people seeking help for symptoms of depression, were recruited sequentially through youth clinics, general practices, and school based	5
65	Mi-seon Kim, 2014	Social Stories (SS): computer-based ss picture symbols system combined with video modelling to teach social communicative skills.	To examine the use of tablet assisted Social StoriesTM intervention for high school students	South Korea	Quantitative	17 years	3	NK	Participants were targeted for intervention according to number of criteria.	4
66	Williams, 2002	CAI: Computer assissted instruction (CAI) includes book and card games scanned into a computer including sounds that children can read similar to [illegible]	To evaluate the progress of eight children in their development of reading skills in two conditions	UK	Quantitative	3-5 years (M = 4.6; SD 1.01)	8	NK	Recruited through the local autism specialist unit within a special school.	3

67	Kobak, 2015	Technology enhanced CBT intervention for the treatment of adolescent depression consisting of (1) online therapist training (2) in-session use of tablets for teaching clients CBT concepts and skills, and (3) text messaging for between session homework reminders and self-monitoring.	To evaluate the feasibility, user satisfaction, and effectiveness of this technology enhanced approach for treating adolescent depression	USA	RCT	12–17 years (M = 15.4; SD = 1.52)	65	89.74	Clinicians who work with depressed adolescents participated in the study. Clinicians were recruited through advertisements in professional journals and through direct mail (i.e., Psychology today listing of clinicians working with depressed adolescents). Each clinician [illegible]	3
68	Fleischman n, 2018	StudyCare Stress is based on the principles of cognitive behavioral therapy and constitutes a modification of Get.On Stress, an online stress management intervention.	To explore the experiences of students participating in an Internet- and App-based stress management intervention originally [illegible]	Germany	Qualitative	20 - 32 years (M= 24; SD= 4)	10	NK	Participants were selected from an existing randomized controlled trial that evaluated the effectiveness of an Internet- and App-based stress training.	5
69	Jones, 2015	TE-HNC (Technology-Enhanced Helping the Noncompliant Child) includes the Helping the Noncompliant Child program, as well as smartphone components that were developed to enhance, rather than replace, clinic-based, therapist guided services based on the applied behaviour analysis model.	To develop and pilot test a technology-enhanced version of one evidence-based Behavioral Parent Training (BPT) program (Helping the Noncompliant Child - HNC) for Disruptive Behavior Disorders (DBDs)	USA	RCT	3-8 years (M= 5.670)	22	83.36	Low-income families in north central North Carolina (NC) were recruited via: (1) Advertisements targeting areas, work places, and retail outlets with an overrepresentation of low-income parents (48% of pilot families); (2) Healthcare, social service, and other agencies that serve low-income families (28% of pilot families); (3) Local schools (19% of [illegible]	1
70	Kennard, 2018	ASAP: As Safe as Possible is a brief App-supported intervention consisting of four modules. The intervention includes a telephone app (BRITE) that promotes emotion regulation and provides access to a personalized safety plan during transition from inpatient care to outpatient care.	To report on a pilot study of an inpatient intervention for suicidal adolescents, As Safe as Possible (ASAP), supported by a smartphone app (BRITE) to [illegible]	USA	RCT	12–18 years (M=15.1;SD= 1.5)	66	81.81	Recruited from the psychiatric inpatient units at two academic medical centers.	3

71	Peters, 2017	Kiss My Asthma: An Asthma Self--Management App for psychological support using self-determination theory.	To examine the psychological experience of asthma as reported by young people and provide examples of how an app for asthma can be designed to	Australia	Qualitative	M=17.8	20	27.10	Conveience sampling via communications sent through social media, university websites, community pharamacies, sporting clubs, high schools nurses, asthma educators, and organizations providing services for	5
72	Edbrooke-Childs, 2019	Power Up is a transdiagnostic and transtherapeutic intervention to promote patient activation and shared decision-making among young people.	To determine the feasibility of undertaking a cluster randomized controlled trial to test the effectiveness of a smartphone app to support patient activation and shared decision making for	UK	Mixed-methods	CAMHS: M=14.66; SD=1.99 Schools: M=16.88; SD=0.68	142	45.07	Participants were recruited through CAMHS and schools.	3
73	Traber-Walker, 2018	Robin, a specific CBT smartphone application to support the therapy of adolescents with attenuated or full-blown psychotic symptoms. The smartphone application targets medication adherence, real-time symptom assessment and provides help coping with symptoms and stressful situations in daily life. The application is only used in combination with psychotherapy in our university	To pilot test a smartphone application to support the therapy of adolescents with attenuated or full-blown psychotic symptoms	Switzerland	Qualitative	14-18 years	7	100.00	NR	4
74	Lattie, 2017	TECH: Teen Enaged in Collaborative Health is a peer-networked internet delivered treatment intervention. CBT-based	To examine the feasibility and acceptability of the intervention program and	USA	Quantitative	14-19 years	40	95.00	Recruitment primarily took place through advertisements on social media (Instagram), as well as through schools	4
75	Ho, 2016	TECH: Teen Enaged in Collaborative Health is a peer-networked internet delivered treatment intervention. CBT-based	To examine whether peer-networked support promotes adherence to BITs	USA	Mixed-methods	15-19 years	13	100.00	Adolescent participants were recruited from schools, community agencies, and word of mouth	3
76	Whitehouse, 2017	TOBY (Therapy Outcomes By You) is an app-based learning curriculum designed for children and parents as a complement to	To test the efficacy of one technology-based early intervention programme	Australia	RCT	M = 3.38; SD = 0.69	80	84.00	NR	4

77	Bekele, 2013	ARIA: Adaptive Robot-Mediated Intervention Architecture involves the child sitting facing the human therapist or the robot administrator in their respective sessions and the human therapist and the robot presented the joint attention task using the hierarchical prompt protocol. A hierarchical prompt protocol was [illegible]	To develop an individualized and adaptive robotic therapy platform and to conduct a usability study that investigates the potential of such a robot-mediated ASD intervention as compared to [illegible]	USA	Quantitative	2–5 years (M= 4.70; SD=0.7)	12	66.70	All participants with ASD were recruited through existing clinical research programs at Vanderbilt University.	3
78	Kumazaki, 2018	CommU is a 304 mm tall robot designed to prmote joint attention in children with ASD.	To test whether the robot is more useful in facilitating joint attention (JA) than a human agent in children with ASD during an [illegible]	Japan	RCT	5–6 years Intervention: M = 5.88; SD= 0.51	68	97.06	Participants were asked to take part in the study at enrollment to the Research Center for Child Mental Development, Kanazawa University.	3
79	Schoenfelder, 2017	The Fitbit Flex & Facebook: collects data through its built-in accelerometer to provide proxy estimates of PA including steps, energy expended, and distance traveled. Users are asked to join a Facebook group to receive information and encouragement and digital badges are earned for [illegible]	To evaluate feasibility and acceptability of an innovative intervention incorporating an mHealth-linked wearable activity tracker (Fitbit Flex) and a Facebook group to increase [illegible]	USA	Mixed-methods	14–18 years (M=15.5;SD = 1.4)	11	100.00	Recruited using flyers through primary care and specialty clinics affiliated with a children's hospital.	5
80	Zhang, 2018	CVE: Collaborative Virtual Environment where 2 geopgraphically distributed users can interact and communicate with each other in a shared environment. Several puzzle games promoting collaborative-communication and collaborative- [illegible]	To examine the feasbility and performance characteristics of a CVE and an associated strategy for quantifying aspects of social communication and [illegible]	USA	Quantitative	10.59-13.89 years	28	100.00	Participants recruited through an electornic recruitment registry accessible to community families. ASD participants recruited from an existing clinical research registry.	3
81	Yuan, 2018	CAVE: Immersive VRE in a four-side Cave Automatic Virtual Environment (CAVE). Participants enter the CAVE individually and navigate the VRE with guidance and support. Six learning scenarious designed for relaxation, four training scenarios and one [illegible]	To examine the efficacy of a VR-enabled training program on emotional and social skills	Hong Kong	Mixed-methods	M=8.86; SD=1.13	72	74.00	Primary school children diagnosed with ASD from mainstream primary schools were recruited.	3
82	Cai, 2013	VDI (virtual dolphin interaction): Virtual Reality - Dolphin-assisted CBT therapy for autism intervention and treatment [illegible]	To present an innovative design and development of a Virtual Dolphinarium for [illegible]	Singapore	Quantitative	6-17 years	15	NK	A random selection of 15 participants from a cohort of 32 children with autism who had taken part in the dolphin- [illegible]	4

83	Gonzales, 2014	ESQYIR (Educating & Supporting inquisitive Youth in Recovery) involves three types of text messaging: daily selfmonitoring texts, a daily wellness recovery tip, and substance abuse education and social support resource information on weekends.	To examine the feasibility of a 12-week mobile-based aftercare intervention for youth transitioning out of community-based substance abuse treatment programs	USA	RCT	12 - 24 years	80	NK	In-person advertisement during treatment groups or by posting brochure/fliers in treatment waiting areas and group rooms.	3
								0.00		
									79.20044776	
