# Supplementary material for: Engaging Children and Young People in Digital Mental Health Interventions: Systematic Review of Modes of Delivery, Facilitators, and Barriers
Source: J Med Internet Res. 2020 Jun 23;22(6):e16317. doi: 10.2196/16317 (PMC7381028; doi:10.2196/16317)
Supplement: Multimedia Appendix 5 [file jmir_v22i6e16317_app5.pdf]

Themes and subthemes encompassing the factors influencing CYP digital MH intervention usage	Sample codes	Summary of supporting evidence (participants quotes/original authors interpretation)	
		Barrier	Facilitator
Intervention-specific influencing factors			
Suitability	Accessibility	Adolescents indicated that they do not use email or computers frequently and that they would access the intervention more regularly if it were delivered via mobile phone, and notifications were provided by SMS messages [1].<br><br>Technical issues [2, 3]<br><br>Failing technology was a problem. Server malfunction without notification was confusing for the participants and led to some chat-sessions being cancelled, shorter than planned or carried out with difficulty [4]<br><br>Computer problems or computer access [5]<br><br>"transportation (getting a ride, driving, taking a bus) to the clinic for the session" to engage in the intervention [6]	Not having to travel to seek help was mentioned as a key motive. Participants also expressed a positive feeling of immediacy in relation to the intervention [4].<br><br>Being able to communicate in the privacy of one's own home was seen as an advantage, alleviating stress and anxiety resulting from face-to-face meetings, social pressure and/or unfamiliar surroundings [4].<br><br>Ability to use at home provided ease of access and avoiding waitlists to support [5].<br><br>CYP suggested advertising DI studies on Facebook and Twitter in addition to formal avenues of advertising [7].[8]<br><br>cCBT makes mental health support more accessible [8].
	Convenience	Lack of time [2, 3, 5, 9, 10]<br><br>Time pressure and inability to integrate the tasks in everyday life. [11]<br><br>Too time-consuming [12]	Participants appreciate flexibility concerning time and place of exercise and relatively little effort needed for treatment completion. They were also more interested in using DIs because of the long waiting periods for face-to-face treatments [11].

			They liked that YouthCHAT kept them busy in the waiting room and gave them time to reflect on their responses, and what to discuss with their clinician [13].<br><br>“the [internet] net’s where we spend all our time anyway” [7]<br><br>Sunday morning was suggested by two interviewees as a suitable time for each weekly module of the programme to become available as “young people check their email and Facebook on a Sunday afternoon” [7]<br><br>The opportunity to use [it] any time and avoiding waitlists [5]
Usability	Difficulty understanding or using the intervention<br><br>Ease of understanding or using the intervention	Problems understanding the task [11]<br><br>Questions were too difficult for some youth [13]<br><br>Participants were confused about how to use the CBT tools: “I don't understand why we are doing that. Like are we supposed to put in everything I do every day? Or just important events? Or just events I want to do?” “I didn't even know you could “like” things.” [1]<br><br>It was difficult for them to find their way around the program [5, 8]<br><br>Participants agreed that the program was ‘a lot of work’ and over a third (35.7%) agreed that the modules were too long. Almost a half (42.8%) of the participants had felt annoyed or frustrated going through the program.	Easy to use with user-friendly reports and straightforward [13].<br><br>Most of the participants considered SPARX-R easy to use, with 71.4% agreeing that the language was easy to understand, 75.0% agreeing that they understood what they were supposed to do on the program, and 71.4% agreeing that their computer skills were adequate for using the program. Additionally, the program lessons ‘made sense’ to them [8].<br><br>The site was easy to use [2, 12, 14]<br><br>Clinicians found the system ‘user-friendly’ [15]<br><br>Participant liked that it was self-paced [16]

		Also the advice provided was too 'hard going' (i.e. difficult to deal with) by focusing unduly on negative aspects of mental health [8].	The necessity of receiving adequate information on the structure of the intervention and the chat-program was brought up, as was secure data transmission. Also making support simple was important [4].<br><br>Both young people and clinicians were positive about the age-appropriateness of the program content [17].<br><br>Participants recommended more development and clarity of goals for the character, as well as feedback on how to manoeuvre the game[18].
Acceptability	Features	All interviewees suggested not having pictures of meditators on the site as it may communicate a religious overtone [7].<br><br>All interviewees advised against the use of the word 'homework' as it may remind participants of their university or school homework and thus be off putting[7].<br><br>Some participants displayed reluctances in wearing the stereotypic viewing goggles for Virtual reality experiences[10].<br><br>The interface was not appealing enough [13]<br><br>Nearly a half (46.5%) of the users indicated that they found the program boring, too easy to play, having too much talk/writing and being too slow [8].	All interviewees were in favour of the information delivery in video format rather than through text or audio. Although suggesting 'as little text as possible' (2–3 sentences in each page) on webpages they asked that teaching material be available as a downloadable PDF file as well as videos. They suggested pictures of young people 'being happy, being active and having fun' perhaps enhanced with online animation. All interviewees preferred a series of short videos each week rather than one long presentation [7].<br><br>Reasons for choosing an Internet-delivered over a face-to-face treatment were mostly related to the perceived advantages of the treatment format itself: its attractive presentation [11]<br><br>Appropriate for their age group and they liked the

		The inability to ask questions via the DI [5]	site design and specific components [1, 12]<br><br>CYP had a desire for the site to have more ways to personalize their experience, such as adding their own photos to the profile page. They indicated that they would like additional ways to interact with other group members, and asked to receive notifications when other participants posted comments. They also wish for the DI to be delivered via mobile phone, and notifications were provided by SMS messages. [1].<br><br>Participants liked the look of AcSPARX-R (46.5%) or that they thought that the program was fun (39.2%). Although more than half (55.5%) of the participants considered the program interesting. More specifically, they liked: 1) the gaming elements, such as customizing your avatar and completing challenges [8].<br><br>Two interviewees encouraged the use of slang and mobile telephone text message language (e.g. abbreviating 'you' to 'u'), especially in headings and emails (even if automated and using a 'no-reply' address), and text message reminders, were thought to be likely to be helpful without being intrusive [7].<br><br>All interviewees agreed that an online forum, which enabled discussion about their programme experiences, was highly desirable and was likely to boost retention significantly [7]

			Rewards were suggested by interviewees to ensure programme completion [7].<br><br>Features such as: profiles, reminders, tracking, social component, emergency support, expert access and information and feedback and motivational features [19]<br><br>Ability to track progress [5]<br><br>There was a wish to be able to vary the form of communication between live meetings, chat sessions, email conversations, video conversations or even group-chat sessions [4].<br><br>Participants suggested incorporating testimonials from success stories [20]<br><br>attractive structure and layout (4210)<br><br>Users generally found the site to be user-friendly and visually pleasing. All participants commented positively about the quality and age appropriateness of videos, images, animations, logo, and “comic book” style illustrations but suggested adding more visual cues for key concepts. Additionally, participants were positive about the adaptive tailoring features of the IRIS and suggested that this was a critical feature for keeping young people engaged in the intervention over time. [17].

			While automated personalization was seen as a strength, youth testers also provided feedback about their desire for control over aspects of program workflow (e.g., wanting to decide for themselves if parents should be notified of intervention progress; ability to see a summary of their progress over time, ability to self-select when and how often reminder emails were sent to them). The most common suggestions for additional personalization included: 1) additional space for expression (e.g., places to write out comments, having an 'other' option included in a list), and 2) additional choices (e.g., less yes/no options or required answers) [17].
Person-specific influencing factors			
Motivation	Motivation	Decreased motivation were was caused the perceived irrelevancy of certain tasks and a disinclination to write. Further, reasons mentioned for less thorough task completion were lack of ideas [11].<br><br>Lack/Loss of interest[3, 5]<br><br>Forgotten its existence [11]<br><br>Avoidance of dealing with one's own eating disorder [9]	Participants expressed being curious to try out the treatment program [4, 11].<br><br>Parents and adolescents in the public health campaign suggested that successful academic performance would be a motivator to use the site [20].
	Helpfulness<br><br>*Usefulness	not finding the resource helpful (2518)<br><br>information being too general (3824)	Perceived as a helpful therapy planning tool with new ideas for therapy and activities [12].<br><br>The majority of participants had a perceived need as they directly linked their participation to university-related problems, referring to stress

			during thesis writing (bachelor's thesis, master's thesis), due to learning problems and to exam anxiety. Additionally perceived usefulness of the intervention to improve time management, or sleep-related complaints [11].<br><br>Perceived helpfulness, particularly around managing emotions[8].<br><br>The perceived ability of the intervention to support or help with issues specifically pertaining to one's diagnosis or daily life problems [4]
Capability	Not feeling well<br><br>*Stressors	The families who discontinued participation cited a major health (e.g., organ transplant) or family (e.g., divorce) stressor necessitating dropout [21].<br><br>Another participant reported feeling stressed by the reminders that were automatically sent if a participant had not logged on during that week [22].<br><br>Physically unwell and unable to attend appointments [3].<br><br>"Experienced a lot of stress in my life" [6]	
Opportunity	Trust	Some participants questioned the validity of the Web site [23].<br><br>Young people may be reluctant to undertake the programme because of concerns about privacy (e.g. when using a family or other public computer [7].<br><br>A visual image of the person on the receiving end, along with meeting and getting acquainted with the coach	Utilising brand names that young people trust, for example, Reachout.com [7].<br><br>The game communicated a sense of recovery and hope for the future [18].<br><br>A visual image of the person on the receiving end, along with meeting and getting acquainted with the coach were important so as to be able to

		were important so as to be able to share innermost thoughts and feelings [4].<br><br>The worry about privacy of personal information [5] or previous bad experience (e.g. at the 3D movies) or uncertainties [10].	share innermost thoughts and feelings. In addition having knowledge of the coaches' competence and knowledge were considered crucial [4]. Similarly, a more transparent/personalized view of the study team (e.g., photos, credentials prominently displayed) and evidence of the program's credibility/usefulness (e.g., testimonials, endorsements, overview of CBT approaches) [17].<br><br>The physician-social worker review group suggested that the established adolescent-social worker relationship may be useful to encourage adolescents to try the website consistently. Additionally, adolescents would be comfortable if their peers recommended the website [20].<br><br>Familiarity with online treatments [11].
	Anonymity<br><br>*privacy		Clinicians felt that YouthCHAT was acceptable to their young patients because it was electronic and reinforced their privacy[13].<br><br>There was very strong agreement in thinking that it was easier to talk about self-harm online to a stranger than to family or friends. Anonymity was an important factor in this [11, 24]<br><br>The anonymity helped with the alleviation of embarrassment [5].
	Connectedness<br><br>Sense of community	Some people felt alone online. Most young people wanted to know that others had the same feelings [24].<br><br>Feelings of being without therapist support [5].	"it was 'nice' to have someone to talk to and that it was good to say what was going on aloud "(albeit in writing). They described a sense of not being alone, and of having a shared

		One patient reported annoyance with the administration of the treatment, specifically citing feeling “forgotten” by the coach due to not receiving a response to a question for over a week [22].<br><br>Participants noted that their desire to post again in the future was reduced when they did not see comments made by others, or they themselves posted comments and did not receive responses [1].	understanding of each other's difficulties. They also compared themselves to others and as a result believed their situation was not as bad as they thought [23].<br><br>Participants agreed that self-harm forums should be moderated and that it was ‘nice’ if moderators got involved and offered support, as opposed to simply ‘policing’ the site [24].<br><br>Greater interactivity than self-help resources [5].

References

1. Ho J, Corden ME, Caccamo L, Tomasino KN, Duffecy J, Begale M, Mohr DC. Design and evaluation of a peer network to support adherence to a web-based intervention for adolescents. Internet Interventions. 2016;6:50-6.
2. Manicavasagar V, Horswood D, Burckhardt R, Lum A, Hadzi-Pavlovic D, Parker G. Feasibility and effectiveness of a web-based positive psychology program for youth mental health: randomized controlled trial. J Med Internet Res. 2014;16(6):e140.
3. Merry SN, Stasiak K, Shepherd M, Frampton C, Fleming T, Lucassen MF. The effectiveness of SPARX, a computerised self help intervention for adolescents seeking help for depression: randomised controlled non-inferiority trial. BMJ. 2012;344:e2598.
4. Sehlin H, Hedman Ahlstrom B, Andersson G, Wentz E. Experiences of an internet-based support and coaching model for adolescents and young adults with ADHD and autism spectrum disorder -a qualitative study. BMC Psychiatry. 2018;18(1):15.
5. Sweeney GM, Donovan CL, March S, Forbes Y. Logging into therapy: Adolescent perceptions of online therapies for mental health problems. Internet Interv. 2019;15:93-9.
6. Crawford EA, Salloum A, Lewin AB, Andel R, Murphy TK, Storch EA. A Pilot Study of Computer-Assisted Cognitive Behavioral Therapy for Childhood Anxiety in Community Mental Health Centers. Journal of Cognitive Psychotherapy. 2013;27(3):221-34.
7. Monshat K, Vella-Brodrick D, Burns J, Herrman H. Mental health promotion in the Internet age: a consultation with Australian young people to inform the design of an online mindfulness training programme. Health Promot Int. 2012;27(2):177-86.

8. Kuosmanen T, Fleming TM, Barry MM. The implementation of SPARX-R computerized mental health program in alternative education: Exploring the factors contributing to engagement and dropout. Children and Youth Services Review. 2018;84:176-84.
9. Fichter MM, Quadflieg N, Nisslmuller K, Lindner S, Osen B, Huber T, Wunsch-Leiteritz W. Does internet-based prevention reduce the risk of relapse for anorexia nervosa? Behav Res Ther. 2012;50(3):180-90.
10. Yuan SNV, Ip HHS. Using virtual reality to train emotional and social skills in children with autism spectrum disorder. London Journal of Primary Care. 2018;10(4):110-2.
11. Fleischmann RJ, Harrer M, Zarski AC, Baumeister H, Lehr D, Ebert DD. Patients' experiences in a guided Internet- and App-based stress intervention for college students: A qualitative study. Internet Interventions. 2018;12:130-40.
12. Whitehouse AJO, Granich J, Alvares G, Busacca M, Cooper MN, Dass A, Duong T, Harper R, Marshall W, Richdale A, Rodwell T, Trembath D, Vellanki P, Moore DW, Anderson A. A randomised controlled trial of an iPad-based application to complement early behavioural intervention in Autism Spectrum Disorder. J Child Psychol Psychiatry. 2017;58(9):1042-52.
13. Goodyear-Smith F, Corter A, Suh H. Electronic screening for lifestyle issues and mental health in youth: a community-based participatory research approach. BMC Medical Informatics and Decision Making. 2016;16(1).
14. Van Voorhees BW, Fogel J, Pomper BE, Marko M, Reid N, Watson N, Larson J, Bradford N, Fagan B, Zuckerman S, Wiedmann P, Domanico R. Adolescent Dose and Ratings of an Internet-Based Depression Prevention Program: A Randomized Trial of Primary Care Physician Brief Advice versus a Motivational Interview. Journal of cognitive and behavioral psychotherapies : the official journal of the International Institute for the Advanced Studies of Psychotherapy and Applied Mental Health. 2009;9(1):1-19.
15. Kobak KA, Mundt JC, Kennard B. Integrating technology into cognitive behavior therapy for adolescent depression: a pilot study. Ann Gen Psychiatry. 2015;14:37.
16. Saekow J, Jones M, Gibbs E, Jacobi C, Fitzsimmons-Craft EE, Wilfley D, Barr Taylor C. StudentBodies-eating disorders: A randomized controlled trial of a coached online intervention for subclinical eating disorders. Internet Interventions. 2015;2(4):419-28.
17. Wozney L, Baxter P, Newton AS. Usability evaluation with mental health professionals and young people to develop an Internet-based cognitive-behaviour therapy program for adolescents with anxiety disorders. BMC Pediatr. 2015;15:213.
18. Olivet J, Haselden M, Piscitelli S, Kenney R, Shulman A, Medoff D, Dixon L. Results from a pilot study of a computer-based role-playing game for young people with psychosis. Early Interv Psychiatry. 2019;13(4):767-72.
19. Peters D, Davis S, Calvo RA, Sawyer SM, Smith L, Foster JM. Young People's Preferences for an Asthma Self-Management App Highlight Psychological Needs: A Participatory Study. J Med Internet Res. 2017;19(4):e113.
20. Sobowale K, Zhou AN, Van Voorhees BW, Stewart S, Tsang A, Ip P, Fabrizio C, Wong KL, Chim D. Adaptation of an internet-based depression prevention intervention for Chinese adolescents: from "CATCH-IT" to "grasp the opportunity". Int J Adolesc Med Health. 2013;25(2):127-37.
21. Jones DJ, Forehand R, Cuellar J, Parent J, Honeycutt A, Khavjou O, Gonzalez M, Anton M, Newey GA. Technology-enhanced program for child disruptive behavior disorders: development and pilot randomized control trial. J Clin Child Adolesc Psychol. 2014;43(1):88-101.
22. Backman A, Mellblom A, Norman-Claesson E, Keith-Bodros G, Frostvittra M, Bölte S, Hirvikoski T. Internet-delivered psychoeducation for older adolescents and young adults with autism spectrum disorder (SCOPE): An open feasibility study. Research in Autism Spectrum Disorders. 2018;54:51-64.

23. Horgan A, McCarthy G, Sweeney J. An evaluation of an online peer support forum for university students with depressive symptoms. Arch Psychiatr Nurs. 2013;27(2):84-9.
24. Jones R, Sharkey S, Ford T, Emmens T, Hewis E, Smithson J, Sheaves B, Owens C. Online discussion forums for young people who self-harm: user views. The Psychiatrist. 2018;35(10):364-8.
